# The anti-tumor and renoprotection study of E-[c(RGDfK)_2_]/folic acid co-modified nanostructured lipid carrier loaded with doxorubicin hydrochloride/salvianolic acid A

**DOI:** 10.1186/s12951-022-01628-x

**Published:** 2022-09-24

**Authors:** Bing Zhang, Ying Zhang, Wenli Dang, Bin Xing, Changxiang Yu, Pan Guo, Jiaxin Pi, Xiuping Deng, Dongli Qi, Zhidong Liu

**Affiliations:** 1 State Key Laboratory of Component-Based Chinese Medicine, Tianjin University of Traditional Chinese Medicine, Tianjin, 301617 China; 2 Haihe Laboratory of Modern Chinese Medicine, Tianjin, 301617 China; 3 Engineering Research Center of Modern Chinese Medicine Discovery and Preparation Technique, Ministry of Education, Tianjin University of Traditional Chinese Medicine, Tianjin, 301617 China; 4 College of Food and Biological Engineering, Xuzhou University of Technology, Xuzhou, 221008 China

**Keywords:** Tumor-targeting, Drug combination, Nephrotoxicity, Creatinine

## Abstract

**Background:**

Poor in vivo targeting of tumors by chemotherapeutic drugs reduces their anti-cancer efficacy in the clinic. The discovery of over-expressed components on the tumor cell surface and their specific ligands provide a basis for targeting tumor cells. However, the differences in the expression levels of these receptors on the tumor cell surface limit the clinical application of anti-tumor preparations modified by a single ligand. Meanwhile, toxicity of chemotherapeutic drugs leads to poor tolerance to anti-tumor therapy. The discovery of natural active products antagonizing these toxic side effects offers an avenue for relieving cancer patients’ pain during the treatment process. Since the advent of nanotechnology, interventions, such as loading appropriate drug combinations into nano-sized carriers and multiple tumor-targeting functional modifications on the carrier surface to enhance the anti-tumor effect and reduce toxic and side effects, have been widely used for treating tumors.

**Results:**

Nanocarriers containing doxorubicin hydrochloride (DOX) and salvianolic acid A (Sal A) are spherical with a diameter of about 18 nm; the encapsulation efficiency of both DOX and salvianolic acid A is greater than 80%. E-[c(RGDfK)_2_]/folic acid (FA) co-modification enabled nanostructured lipid carriers (NLC) to efficiently target a variety of tumor cells, including 4T1, MDA-MB-231, MCF-7, and A549 cells in vitro. Compared with other preparations (Sal A solution, NLC-Sal A, DOX solution, DOX injection, Sal A/DOX solution, NLC-DOX, NLC-Sal A/DOX, and E-[c(RGDfK)_2_]/FA-NLC-Sal A/DOX) in this experiment, the prepared E-[c(RGDfK)_2_]/FA-NLC-Sal A/DOX had the best anti-tumor effect. Compared with the normal saline group, it had the highest tumor volume inhibition rate (90.72%), the highest tumor weight inhibition rate (83.94%), led to the highest proportion of apoptosis among the tumor cells (61.30%) and the lowest fluorescence intensity of proliferation among the tumor cells (0.0083 ± 0.0011). Moreover, E-[c(RGDfK)_2_]/FA-NLC-Sal A/DOX had a low level of nephrotoxicity, with a low creatinine (Cre) concentration of 52.58 μmoL/L in the blood of mice, and no abnormalities were seen on pathological examination of the isolated kidneys at the end of the study. Sal A can antagonize the nephrotoxic effect of DOX. Free Sal A reduced the Cre concentration of the free DOX group by 61.64%. In NLC groups, Sal A reduced the Cre concentration of the DOX group by 42.47%. The E-[c(RGDfK)_2_]/FA modification reduced the side effects of the drug on the kidney, and the Cre concentration was reduced by 46.35% compared with the NLC-Sal A/DOX group. These interventions can potentially improve the tolerance of cancer patients to chemotherapy.

**Conclusion:**

The E-[c(RGDfK)_2_]/FA co-modified DOX/Sal A multifunctional nano-drug delivery system has a good therapeutic effect on tumors and low nephrotoxicity and is a promising anti-cancer strategy.

**Graphical Abstract:**

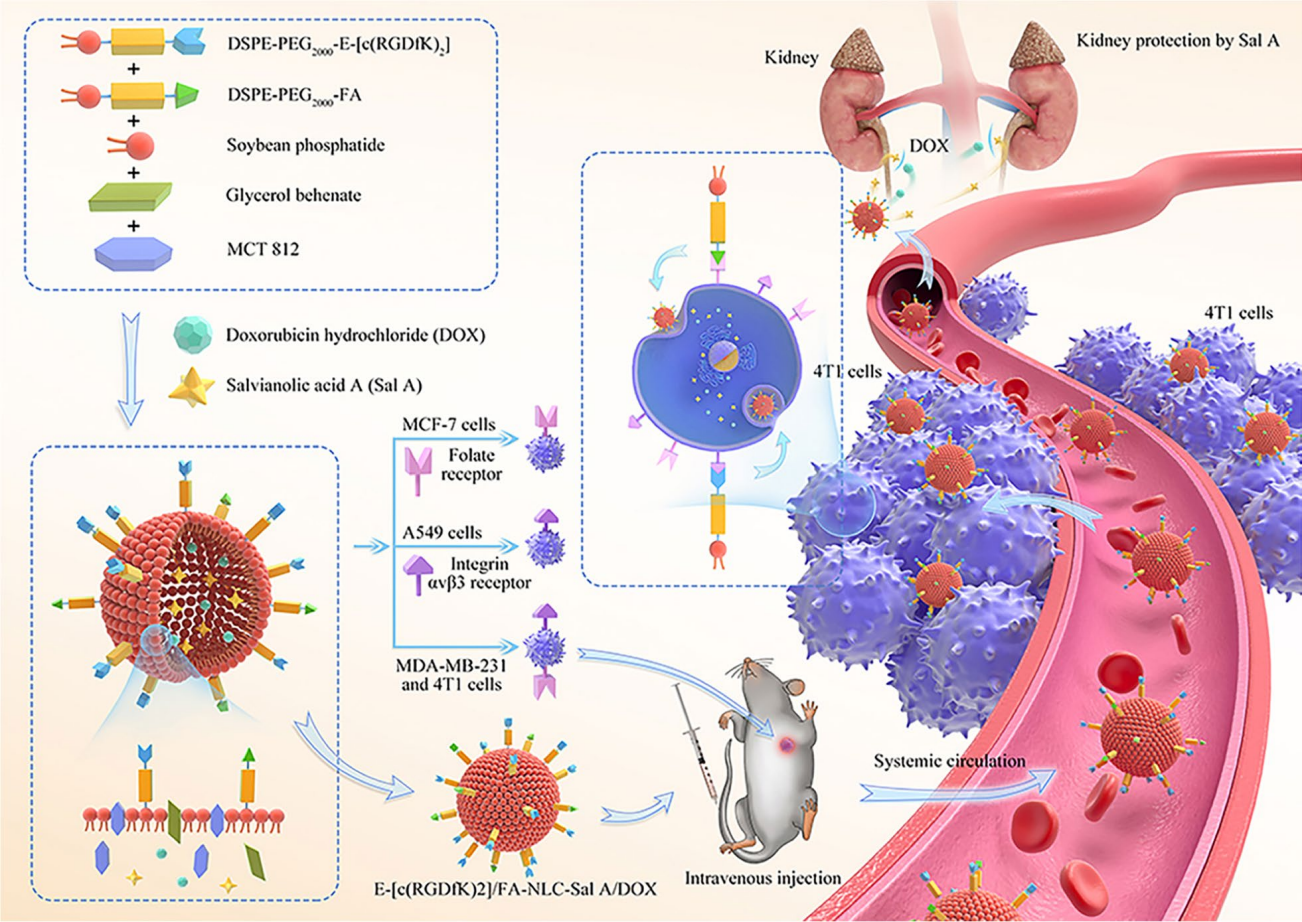

**Supplementary Information:**

The online version contains supplementary material available at 10.1186/s12951-022-01628-x.

## Introduction

Cancer is a grave disease associated with high morbidity and mortality, and threatens human health and life [1]. The clinical treatment of cancer mainly includes surgical resection, radiation therapy, and chemotherapy. During chemotherapy, cytotoxic effects of antineoplastic agents are mediated by a variety of mechanisms, leading to disrupted DNA replication, inhibition of RNA-dependent DNA synthesis, cell-cycle arrest, DNA damage, and cell death [2–5]. Conventional chemotherapy has a long history of being used to treat cancer and can significantly prolong patient survival [6]. Unfortunately, during chemotherapy, problems, such as poor tumor targeting by drugs and their toxicity to other normal organs and tissues have arisen [7, 8]. These problems severely restrict the clinical application of chemotherapy, resulting in unsatisfactory anti-tumor effect and poor patient tolerance [9].

Studies on tumors have found that tumor cells take up substances required for their growth from the tumor environment, including folic acid (FA) [10], peptides containing tripeptide of Arg-Gly-Asp (RGD) [11] and so on. The cellular uptake process is highly dependent on some receptors on the surface of tumor cells that are overexpressed as compared to normal cells, such as FA receptors [12], α_v_β_3_ receptors [13], etc. Further research revealed that these receptors are expressed at different levels on the surface of different tumor cells. For example, human lung cancer cell A549 expresses high levels of α_v_β_3_ receptors but almost no FA receptors [14], and human breast cancer cell MCF-7 expresses high levels of FA receptors on the cell surface, but hardly expresses α_v_β_3_ receptors [15]. The difference in the expression of receptors on the surface of these tumor cells results in differences in the affinity of different types of tumor cells for different ligands.

Inspired by the ligand-receptor binding phenomenon that occurs on the surface of tumor cells, scientists believe that the ligands required by these tumor cells can serve as the guides of anti-tumor drugs, precisely mediating the entry of anti-tumor drugs into tumor cells [16, 17]. In view of this, a novel tumor treatment strategy has been proposed: integrating anti-tumor drugs with ligands required by these tumor cells into a macromolecule, to improve the efficiency of anti-tumor drug entry into tumor cells by taking advantage of these ligands. For example, coupling doxorubicin and FA through PEG can significantly improve the anti-tumor effect of doxorubicin [18]. Although this strategy works well for anti-tumor therapy, unfortunately, it is difficult to link multiple ligands to drugs simultaneously to deal with different receptor expression levels on the surface of different types of tumor cells, which limits further research and development of this strategy.

The development of nanotechnology in recent years has provided another platform for the combination use of drugs and ligands [19], and one of these outstanding platforms is the nanostructured lipid carriers (NLC) [20, 21]. NLC are composed of solid and liquid lipids [22], and have the advantages of long-term biocompatibility, easy surface modification, and high drug encapsulation [23]. Through surface modification, nanoparticles can protect drugs from being absorbed by reticuloendothelial cells and prevent high concentrations of drugs from accumulating in organs, such as the liver, kidney, and spleen [24]. The systemic toxicity of the loaded drug is reduced through the high aggregation of nanoparticles at the tumor site and efficient drug uptake [25–27].

The easy modification of NLC enables simultaneous linking of a variety of tumor-targeting ligands on its surface [28]. The dual tumor-targeted modified carrier has better targeting functions against specific tumor cells. For example, mesoporous silica nanoparticles modified with RGD peptide and FA are used to deliver paclitaxel; the preparation has excellent in vitro inhibition of human breast cancer cells MCF-7 [29]. However, the inherent cytotoxicity of mesoporous silica nanoparticles is a challenge since it can damage normal organs [30–32]. Therefore, a better choice would be a non- or low-cytotoxicity carrier, such as NLC [33, 34].

In the process of targeting modified carriers with RGD peptides, another consideration is the type of RGD peptide. RGD peptides possessing better affinities will impart the carriers with stronger tumor-targeting abilities [35]. Our previous study found that compared with c(RGDfK), NLC modified with E-[c(RGDfK)_2_] has better tumor-targeting properties against mouse breast cancer cell 4T1 [36], however, the tumor-targeting of NLC co-modified with FA and E-[c(RGDfK)_2_] has not been investigated.

The application of NLC modified with tumor-targeting ligands can avoid the serious toxicities and side effects caused by the accumulation of anti-tumor drugs in normal organs [37]. Unfortunately, the anti-tumor drugs loaded in NLC still inevitably produce low toxicity and side effects in organs [38–40]. To counteract the toxicity of anticancer drugs to normal tissues, we use corresponding antagonists [41]. For example, free DOX damages normal kidney cells through mitochondrial oxidative damage caused by production of reactive oxygen species (ROS) [42]. Sal A, as a natural ROS scavenger, has been shown to antagonize the DOX-induced renal cell damage [43]. Notably, Sal A can also inhibit nucleoside transport in tumor cells and enhance the efficacy of anti-tumor drugs during the process of chemotherapy [44]. Although the combined use of free DOX and free Sal A can reduce the nephrotoxicity of DOX [43], DOX in free drug form cannot be efficiently taken up by tumor cells, thereby impeding its anti-tumor effect [45]. Therefore, it is necessary to co-load DOX and Sal A in a tumor-targeting drug delivery system to study the anti-tumor effect and organ toxicity of this drug combination in the drug delivery system.

In this experiment, we constructed E-[c(RGDfK)_2_]/FA-modified NLC loaded with DOX/Sal A. Here, NLC was double-modified with E-[c(RGDfK)_2_] and FA, which ensured that the preparation had an excellent tumor-targeting effect on various tumor cells (4T1 cells, MDA-MB-231 cells, MCF-7 cells, and A549 cells). DOX and Sal A were innovatively co-loaded in NLC to enhance the anti-tumor effect of DOX while at the same time reducing its nephrotoxicity. Our study provides a valuable reference for future research on tumor-targeting double-modified NLC loaded with various drugs.

## Materials and methods

### Materials

Doxorubicin hydrochloride (DOX) and salvianolic acid A (Sal A) were purchased from Shanghai Yuanye Biotechnology Co., Ltd., China, and their purities were ≥ 98%; doxorubicin hydrochloride for injection (DOX Injection) was purchased from Shanxi Pude Pharmaceuticals Co., Ltd., China; DSPE-PEG_2000_-Folate and DSPE-PEG_2000_-COOH were provided by Nanosoft Biotechnology LLC, NC, USA; *N*-hydroxysuccimide (NHS) of purity ≥ 98% was supplied by Acros Organics Inc., USA; 1-ethyl-3-(3-dimethy-laminopropyl) carbodiimide (EDC) of purity ≥ 98.5% was obtained from Shanghai Civi Chemical Technology Co., Ltd., China; E-[c(RGDfK)_2_] were synthesized by Shanghai Qiangyao Biotech Co., Ltd., China; Dialysis membranes bags of ≥ 2000 Da were also purchased from Shanghai Yuanye Biotechnology Co., Ltd., China; Soybean phosphatide was achieved from Shanghai A.V.T. Pharmaceutical Technology Co., Ltd Co., Ltd., China; Polyethylene (40) stearate (Myrj 52) was brought from Sigma-Aldrich LLC., USA; MCT 812 (Miglyol^®^ 812) was purchased from Beijing Feng Li Jing Qi Trading Co., Ltd. China; Glycerol behenate (Compritol^®^ 888 ATO) were procured from Gattefossé Trade Co., Ltd., France; Chloroform (analytical grade) was purchased from Tianjin Damao Chemical Reagent Co., Ltd.; Acetonitrile, methanol, ethanol and phosphoric acid (HPLC grade) were obtained from Thermo Fisher Scientific Co., Ltd.; PBS, RPMI 1640 medium, MEM medium, DMEM medium, Fetal Bovine Serum (FBS), 96-well black basal cell culture plate were purchased from Corning Inc., USA; DMSO and Coumarin-6 were provided by Sigma-Aldrich, China; 0.25% trypsin + 0.02% EDTA and double antibiotics (10,000U penicillin streptomycin) was supplied by Gibco Inc., USA; Hoechst 33342 was obtained from Hoechst AG Inc., Germany; Dir fluorescent dye was achieved from AAT Bioquest, Inc., USA; Mitotracker Deep Red FM and Rhodamine123 were purchased from Invitrogen Inc., USA; anti-Ki67 and TUNEL assay kit were brought from Abcam (Shanghai) Trading Co., Ltd., China; paraformaldehyde (4%) was obtained from Solarbio Science and Technology Co., Ltd., China; Hematoxylin was procured from Wuhan Biotechnology Co., Ltd., China; Creatinine kit was purchased from Nanjing Jiancheng Bioengineering Institute, China; MDA-MB-231 cell lines, 4T1 cell lines, MCF-7 cell lines, A549 cell lines and HEK293 cell lines were purchased from Cell Bank of the Chinese Academy of Sciences, China.

### Cell culture

MDA-MB-231 cell lines, 4T1 cell lines and MCF-7 cell lines were cultivated in a complete medium consisted of 90% DMEM basal medium, 10% FBS, streptomycin (100 units/mL) and penicillin (100 units/mL). A549 cell lines were cultured in complete medium consisted of 90% RPMI 1640 basal medium, 10% FBS, streptomycin (100 units/mL) and penicillin (100 units/mL). HEK293 cell lines were cultivated in a complete medium consisted of 90% MEM basal medium, 10% FBS, streptomycin (100 units/mL) and penicillin (100 units/mL). The five cell lines were maintained in a constant temperature incubator at 37 °C under a 5% CO_2_ atmosphere.

### Animals

Healthy female BALB/c nude mice, 6–8 weeks, weight 17–19 g, were obtained from Beijing HFK Bioscience Co., Ltd. These mice were fed in the animal house at the temperature of 25 ± 2 °C and the relative humidity of 50 ± 2% in Chinese Academy of Medical Sciences (Tianjin, China). Before the experiment began, these mice were raised for a week to adapt to the new external environment. All experiments on the mice were carried out complying with relevant regulations of animal experiments handling issued by the Animal Research Center of Tianjin University of Traditional Chinese Medicine. All operations were authorized by the Animal Ethics Committee of Tianjin University of Traditional Chinese Medicine (Document number: TCM-LAEC2020023).

### Conjugation and characterization of E-[c(RGDfK)_2_] peptides with DSPE-PEG_2000_-COOH

We synthesized DSPE-PEG_2000_-E-[c(RGDfK)_2_] by forming an amide bond between the carboxylic group of DSPE-PEG_2000_-COOH and the amine group of the E-[c(RGDfK)_2_] peptides. We employed EDC/NHS as the carboxylic group’s activator with the molar ratio of DSPE-PEG_2000_-COOH: E-[c(RGDfK)_2_]: EDC: NHS as 1:1:2:2.

DSPE-PEG_2000_-COOH, EDC and HNS were stirred at 50 rpm at room temperature for at least 30 min to be dissolved in PBS (pH = 7.4). E-[c(RGDfK)_2_] was dissolved in another ampoule of PBS (pH = 7.4) to be added drop by drop into the above activated carboxylic group solution, and the mixed solution was stirred at 50 rpm, 25 °C for 24 h. Then we purified the mixed solution with the membrane dialysis bag (cutoff MW of 2000 Da) in distilled water for 36 h to remove the uncombined raw material (DSPE-PEG_2000_-COOH, E-[c(RGDfK)_2_], EDC and HNS). The freeze-drying method was utilized to process the purified solution to obtain the dried powder of DSPE-PEG_2000_-E-[c(RGDfK)_2_]. DSPE-PEG_2000_-COOH, E-[c(RGDfK)_2_] and DSPE-PEG_2000_-E-[c(RGDfK)_2_] were characterized using Maldi-TOF–MS to verify the success of the conjugation reaction.

### Preparation of NLC

The NLCs in our study were prepared by the emulsification-solvent evaporation method. DOX, Sal A, and Myrj 52 were dissolved in deionized water and this constituted the aqueous phase; here, the mass ratio of DOX/Sal A was about 1/10. Lecithin (or together with DSPE-PEG_2000_-E-[c(RGDfK)_2_]) was dissolved in ethanol; Compritol 888 ATO and MCT 812 (or along with DSPE-PEG_2000_-FA) were dissolved in chloroform, and the mixture of these two organic solutions constituted the oil phase. The oil phase was quickly added into the water phase, stirred at 75–80 °C at 800 rpm. After the solution system was clarified, the stirring was continued at a constant temperature of 75–80 °C and at 100 rpm for 1 h.

### Characterization of NLC

#### Determination of particle size and zeta potential

Particle size, polydispersity index (PDI) and ζ potential (ζ-P) of the NLC-Sal A/DOX, FA-NLC-Sal A/DOX, E-[c(RGDfK)_2_]-NLC-Sal A/DOX and E-[c(RGDfK)_2_]/FA-NLC-Sal A/DOX were determined by a Zeta-sizer (Nano-ZS; Malvern Instruments Ltd., UK). The preparations were measured for their ζ-P, and then the stock solutions were diluted 5 times with deionized water to determine the particle size and PDI. All the processes were carried out at room temperature, all samples were tested three times.

#### Differential scanning calorimetry (DSC)

A thermal analyzer (Jade DSC, PerkinElmer Inc. USA) was used to determine the powder samples of raw materials and all the preparations to characterize the physical state of DOX and Sal A in each group of NLC. The test temperature ranged from 30 to 400 °C, with the samples heated at a constant rate of 10 °C/min in a nitrogen atmosphere.

#### Transmission electron microscopy (TEM)

TEM (Tecnai G2 F20, FEI Co., USA) was employed to observe NLC preparations’ morphology. The preparations were diluted with deionized water and dropped onto a copper grid to form a film, negatively stained with 2% phosphotungstic acid, and then dried before being observed using TEM.

#### X-ray diffraction (XRD)

X-ray diffraction (XRD) images were obtained using an X-ray diffractometer (Rigaku D/max 2500/PC, Japan) with a Cu target/graphite monochromator to select the Cu–Kα radiation as an incident beam (λ = 1.54 Å) over a scanning angle range from 5° to 90° with a voltage of 40 kV and a current of 200 mA, operated at 5° min^−1^ in 1 s.

#### Determination of encapsulation efficiency

The unencapsulated DOX and Sal A in the NLC preparations were obtained by centrifugation at 4000 rpm for 20 min using ultrafiltration centrifuge tubes (Amicon ultra, MWCO 30 kDa; Millipore Company, USA) at 4000 rpm for 20 min, and then the ultra-filtered solutions were analyzed using HPLC–UV to acquire the concentrations of the free DOX and Sal A.

The NLC solutions were mixed with the appropriate volume of methanol, and the NLCs were demulsified by ultrasonically for 15 min. The obtained dispersion was centrifuged at 12,000 rpm for 20 min to obtain the supernatant, which was then analyzed by the HPLC–UV for the total weights of DOX and Sal A in the NLC preparations. Efficiency (EE%) was calculated by using the following formula:

$${\text{EE}}{\%} = \frac{{\text{W}_{\text{encapsulated}}}}{{\text{W}_{\text{total}}}} = \frac{{\text{W}_{\text{total}} - {\text{W}}_{\text{free}}}}{\text{W}_{\text{total}}} \times 100\%$$

Here, W_total_ and W_free_ are the total DOX (or Sal A) content and the free DOX (or Sal A) weight in the preparations, respectively.

The DOX and Sal A concentrations (μg/mL) in preparations were measured using a high-performance liquid chromatography system (LC-20AT, liquid chromatograph, Shimadzu Co., Kyoto, Japan) equipped with a dual-wavelength detector. The column used was an Agilent Zorbax SB-C18 (4.6 × 250 mm, 5 μm). The chromatographic separation was operated using gradient elution with a total run time of 20 min at 30 °C and a flow rate of 1 mL/min. Mobile phase A was acetonitrile, and mobile phase B was aqueous phosphoric acid (0.2%, v/v). The gradient elution procedure was as follows: 7.5–9 min, 38–95%A; 9–11 min, 95%min; 11–12 min, 95–25%A; 12–20 min, 25%A. The wavelengths used were 254 nm for DOX [46, 47] and 285 nm for Sal A [48], and the injected volume of the sample was 10 µl. The method’s linear range was 0.47–119.60 μg/ml for DOX and 0.40–103.50 μg/ml for Sal A.

The linear regression equation of DOX was y = 26746x − 2693.2 (correlation coefficient R^2^ = 0.9999) and the one of Sal A was y = 31155x − 4695.9 (correlation coefficient R^2^ = 0.9998). Intra- and inter-day precisions of DOX were within 0.27 to 1.56% and 1.25 to 2.95%. Intra- and inter-day precisions of Sal A were within 0.38 to 0.96% and 0.49 to 2.98%.

### In-vitro cellular uptake studies in tumor cells

Coumarin-6 (C6) was used as the fluorescent probe to label NLC-C6, FA-NLC-C6, E-[c(RGDfK)_2_]-NLC-C6 and E-[c(RGDfK)_2_]/FA-NLC-C6. These NLCs labeled with C6 were prepared using a method similar to that for NLC-Sal A/DOX (here, the C6 was dissolved in chloroform), and C6 was dissolved in DMSO for the C6 stock solution. The concentrations of C6 were determined by HPLC. The above relevant preparations were diluted by the culture medium to obtain a C6 concentration of 0.05 μg/mL, and the blank NLCs were diluted 1000 times to reach non-toxic concentrations (which had been verified by experiments) to the tumor cell lines. The experiments in this section were performed on four types of cancer cells (MDA-MB-231, MCF-7, A549, and 4T1). The fluorescence intensity of these labeled cells was quantitatively analyzed by an Operetta™ high-content system (Perkin Elmer, USA).

To evaluate the uptake of the preparations by the four kinds of cells, MDA-MB-231 cells (6000 cells/well), MCF-7 cells (10,000 cells/well), A549 cells (7000 cells/well), and 4T1 cells (4000 cells/well) were seeded in 96-well black plates. They were cultured in an incubator at a constant temperature of 37 °C under a 5% CO_2_ atmosphere. After incubating for 24 h, Hoechst 33342 was used to stain the cell nucleus by incubating it for 30 min with the cells, and then we washed the wells with phosphate-buffered saline (PBS) thrice to remove the free Hoechst 33342. C6 solution, NLC-C6, FA-NLC-C6, E-[c(RGDfK)_2_]-NLC-C6, and E-[c(RGDfK)_2_]/FA-NLC-C6 were added into the wells and incubated with the cells for 8 h [49, 50], respectively. Then, we washed away the free C6 solution and related NLC-C6 with PBS (thrice) and added 100 μL of the corresponding basal medium into each well.

To verify that the active targeting effect of E-[c(RGDfK)_2_]/FA-NLC is triggered by their specific binding between the α_v_β_3_ receptors and E-[c(RGDfK)_2_], FA receptors and FA, we used excess free E-[c(RGDfK)_2_] and free FA to inhibit the binding of E-[c(RGDfK)_2_] and FA to their receptors [51, 52]. The appropriate weights of FA and E-[c(RGDfK)_2_] were diluted to a concentration of 1 μg/mL in the corresponding basal medium. MDA-MB-231 cells (6000 cells/well), MCF-7 cells (10,000 cells/well), A549 cells (7000 cells/well), and 4T1 cells (4000 cells/well) were seeded into 96-well black plates. After 24 h of incubation, Hoechst 33342 was used to stain the cell nucleus by incubating it for 30 min with the cells, and then we washed the wells with phosphate-buffered saline (PBS) to remove the free Hoechst 33342. The free FA solution and free E-[c(RGDfK)_2_] solution were incubated with the four types of cells for 60 min, respectively. We washed away the free FA and E-[c(RGDfK)_2_] with PBS before adding the diluted E-[c(RGDfK)_2_]/FA-NLC-C6 to the treated and untreated wells of the corresponding cells and incubating for 8 h. The free E-[c(RGDfK)_2_]/FA-NLC-C6 was washed away with PBS before adding 100 μL of the corresponding basal medium to each well.

The processed 96-well plates were loaded into the Operetta™ high-content system. The entire process was protected from light. The excitation wavelength of the nuclear dye Hoechst 33342 was set at 346 nm and an emission wavelength of 460 nm. The excitation wavelength of Cou-6 was 360 nm, and the emission wavelength was 477 nm. Quantitative analysis of the fluorescence intensity was carried out using the software of Perkin Elmer Harmony in version 3.5.1.

### In-vitro renal cell viability and mitochondrial characterization

The attenuating effect of Sal A on DOX-induced renal cytotoxicity was tested using the CCK-8 kit. DOX stock and Sal A stock were prepared using DMSO and MEM media, respectively. The DOX solution used in the experiment was diluted with MEM medium. The DOX/Sal A solution was obtained as follows: the DOX stock solution and the Sal A stock solution were diluted with MEM medium to twice the required concentration, and then they were mixed evenly in a ratio of 1:1.

HEK293 cells (8000 cells/well, 100 μL) were seeded in a 96-well plate and cultured at 37 °C and 5% CO_2_. After 24 h incubation, the medium was discarded, 10% CCK-8 solution was added into the wells, and then the cells were incubated at 37 °C, 5% CO_2_ for 1 h. A microplate reader (SPARK, TECAN, Austria) was employed to detect the OD450 value, and the cell viability was calculated to evaluate the effect of Sal A antagonizing DOX renal cytotoxicity in vitro.

Determination of mitochondrial mass and membrane potential in HEK293 cells was conducted by flow cytometry (BD Biosciences). Formulations for experiments were prepared as previously described, including DOX solution, DOX/Sal A solution, DOX/Sal A-NLC, FA-DOX/Sal A-NLC, E-[c(RGDfK)_2_]-DOX/Sal A-NLC and FA/E-[c(RGDfK)_2_]-DOX/Sal A-NLC. The formulation was diluted with MEM medium to the desired concentration (0.281 μg/mL of DOX and 2.81 μg/mL of Sal A). HEK293 cells (40,000 cells/well, 2.5 mL) were seeded in 6-well plates, and they were cultured at 37 °C and 5% CO_2_. After 24 h, the medium was removed, 2.5 mL prepared formulations were added into each well and discarded after 4 h and 24 h, respectively.

Mitotracker Deep Red FM and Rhodamine 123 were used as fluorescent dyes to measure mitochondrial mass and membrane potential, respectively, and Hoechst 33342 was used to locate the nucleus. Added 1.0 mL of Hoechst 33342 (1.67 μg/mL) and 1.0 mL of Mitotracker Deep Red FM (0.08 μmol/L) prepared with MEM basal medium to each well, and the cells were incubated at 37 °C and 5% CO_2_ for 30 min in the dark. The medium was discarded, and PBS of 4 °C was used to wash the cells, 1.0 mL of MEM basal medium containing 1.26 μg/mL of Rhodamine123 was added into each well, and then the cells were incubated at 37 °C and 5% CO_2_ for 30 min in the dark. The medium was discarded, and PBS of 4 °C was used to wash the cells twice. 1.0 mL PBS was added into each well. Fluorescent staining was visualized by the Operetta^®^ High Content Imaging system (Perkin Elmer, Rodgau, Germany) at 400× magnification.

The cell staining process was repeated, then 0.5 mL of trypsin was added to each well and incubated for 30 s, 1.0 mL of culture medium was added into the wells to stop digestion, the mixture was centrifuged, and the harvest cells were washed twice using PBS of 4 °C, finally the cells were resuspended with 0.5 mL of PBS. The corresponding three channels (MitoTracker RL1, Hoechst VL1, Rhodamine BL1) of Hoechst 33342, Mitotracker Deep Red FM, and Rhodamine 123 for fluorescence quantification were selected on the flow cytometer (BD Biosciences). The results were used to evaluate the changes of mitochondrial mass and mitochondrial membrane potential in HEK293 cells. And per data acquisition was carried out as collecting 10,000 events.

### In-vivo bio-distribution study

DiR was employed as the fluorescence probe, which was loaded in the NLCs. The fluorescence of DiR was detected with an In Vivo Imaging System (IVIS) to trace the in vivo tumor and tissue distribution of NLCs in BALB/c nude mice with 4T1.

4T1 cells in the logarithmic growth phase were gathered and then washed with RPMI medium 1640 to remove the serum. The collected 4T1 cells were resuspended in PBS at a density of 1 × 10^7^ cells/mL. The PBS suspension of 4T1 cells (0.2 mL) was injected in the right armpit of female BALB/c nude mice (17–19 g) When the tumors were around 100–200 mm^3^ (V = ab^2^/2, a = length, b = width), the mice were randomly assigned to seven groups (n = 6 per group), as follows: normal saline, DiR solution, NLC-DiR, PEG_2000_-NLC-DiR, E-[c(RGDfK)_2_]-NLC-DiR, FA-NLC-DiR, and E-[c(RGDfK)_2_]/FA- NLC-DiR. All preparations were administered via the tail vein injection. The dosage of DiR in each group was 0.5 mg/kg, and the injection volume of normal saline was 0.2 mL.

At four time points of 1 h, 4 h, 8 h and 24 h after administration, the nude mice were anesthetized by isoflurane, then the mice were placed in the IVIS, and the fluorescence distribution of DiR in each mouse was observed. Twenty-four hours after the injection, seven tumor-bearing nude mice from the seven groups (one per group) were sacrificed. The heart, liver, spleen, lung, kidney and tumor were collected and detected in IVIS for the distribution of NLCs in organs and tumors. The excitation wavelength of DiR was set at 748 nm, and the emission wavelength was set at 780 nm. Fluorescence intensity (FI) in the pictures was measured by ImageJ software.

### In-vivo antitumor effects

#### Establishing the breast cancer model

To obtain tumor-bearing mice, we injected a PBS suspension (0.2 mL) of 4T1 cells at a density of 1 × 10^7^ cells/mL into the right armpit of female BALB/c mice (16–20 g). When the tumor volume increased to around 50 mm^3^, the mice were randomly divided into nine groups as follows: normal saline, adriamycin hydrochloride injection (positive drug), Sal A solution, Sal A-NLC, DOX solution, Sal A/DOX solution, DOX-NLC, Sal A/DOX-NLC, E-[c(RGDfK)_2_]/FA-Sal A/DOX-NLC. The volume of tail vein injection in each group was 0.2 mL. The dosage of one administration for DOX in each mice was 2 mg/kg, and the dosage of Sal A was 20 mg/kg, which was injected once every two days for a total of 6 administrations.

### In-vivo study of creatinine (Cre) concentration, alanine aminotransferase (ALT) activity and creatine kinase (CK) activity

Twenty-four hours after the last administration, blood was collected from the cheek venous plexus of mice. The corresponding plasma was obtained after centrifugation in heparin sodium anticoagulation tubes. The plasma creatinine (Cre) concentration, alanine aminotransferase (ALT) activity, and creatine kinase (CK) activity were measured with the corresponding kits from the Nanjing Institute of Biological Engineering (Nanjing, China) following the manufacturer’s protocols to evaluate the damage caused by the preparations to the kidney, liver, and muscle cells.

### In-vivo antitumor activity study

The tumor volume was measured with a Vernier caliper every 2 days, and the mouse weight was determined with an electronic balance. Blood was collected from the mice after 6 administrations, the mice were sacrificed, and tumors and organs were collected and weighed to calculate the relative tumor volume (RTv), relative tumor weight (RTw), tumor volume inhibition rate (IRv), tumor weight inhibition rate (IRw), and organ coefficient (Oc) to evaluate the anti-tumor effect and the effect on the various organs of the preparations. The following are the parameter calculation formulas:

$${\text{RTv}} = \frac{{{\text{V}}_{{\text{T}}} }}{{{\text{V}}_{{\text{M}}} }} \times 100$$

Here, V_T_ and V_M_ are the tumor volumes of the treated groups and the average tumor volume of the normal saline group, respectively, at the end of the treatment.

$${\text{RTw}} = \frac{{{\text{W}}_{{\text{T}}} }}{{{\text{W}}_{{\text{c}}} }} \times 100$$

Here, W_T_ and W_c_ are the treated groups’ tumor weights and the average tumor weight of the normal saline group, respectively, at the end of the treatment.

$${\text{IRv}} = \frac{{{\text{V}}_{{\text{C}}} - {\text{V}}_{{\text{T}}} }}{{{\text{V}}_{{\text{C}}} }} \times 100$$

Here, V_C_ and V_T_ are the tumor volumes of the normal saline group and the tumor volumes of treated groups, respectively, at the end of the treatment.

$${\text{IRW}} = \frac{{{\text{W}}_{{\text{C}}} - {\text{W}}_{{\text{T}}} }}{{{\text{W}}_{{\text{C}}} }} \times 100$$

Here, W_C_ and W_T_ are the average tumor weight of the normal saline group and the tumor weights of treated groups, respectively, at the end of the treatment.

$$\text{Oc}=\frac{{\text{Wo}} \; \text{(mg)}}{{\text{Wb}} \;\text{(g)}}\times 100$$

Here, W_o_ and W_b_ are the mice’ organ weights and the body weights, respectively, at the end of the treatment.

### Histological examination

Histological examinations (HE) of mouse organs harvested were performed to assess microscopic damage. The isolated organs were fixed in 4% paraformaldehyde and paraffin-embedded. The paraffinized organs were cut into 4 μm thick slices. The organ sections were deparaffinized in xylene and dehydrated in graded alcohol after being placed on glass slides. Hematoxylin and eosin were then used to stain the organ sections (H&E). Finally, all organ sections were observed and photographed at a magnification of 200 using a Nikon Eclipse CI microscope equipped with an imaging system of NIKON digital sight DS-FI2 (Tokyo, Japan).

### Immunohistochemical analysis

Immunohistochemistry (IHC, including Ki67 and TUNEL) was done to investigate the effects of preparations on tumor cell proliferation and apoptosis, respectively.

After washing the sections with gradient ethanol, the slices were retrieved with EDTA antigen retrieval buffer. After treating the sample with 3% hydrogen peroxide solution (hydrogen peroxide:deionized water = 1:9), they were sealed with 3%BSA. The sections were sequentially treated with the primary antibody (Ki67) and the HRP-labeled secondary antibody. Then, the slices were dealed with diaminobenzidine (DAB), followed by counterstaining with Harris Hematoxylin. The samples were dehydrated with gradient ethanol-xylene.

The sections were observed and photographed. Image pro plus 6.0 (Media Controlnetics, Inc., Rockville, MD, USA) was used to analyze the pictures by measuring the average optical density (MOD) of Ki67 to evaluate the tumor cells’ proliferation.

After washing the sections with gradient ethanol, they were covered with proteinase-K solution (proteinase-K:PBS = 1:9). After rupturing the membrane, a mixture of reagent 1 (TdT) and reagent 2 (dUTP) was added according to 2:29 (v/v). The 4ʹ,6-diamidino-2-phenylindole (DAPI) solution was then added dropwise onto the samples, and finally, the anti-fluorescence quencher was used to seal the sections. Images were collected and observed under an inverted fluorescence microscope. Image pro plus 6.0 (Media Controlnetics, Inc., Rockville, MD, USA) was used to measure the percentage of positive apoptotic cells.

### Statistical analysis

All result data are expressed as mean ± standard deviation. One-way ANOVA analysis was performed by SPSS 22. The *p* value < 0.05 is considered statistically significant. The figures are drawn by GraphPad prism 8.0.1.

## Results and discussion

### Preparation and characterization of NLC

#### Conjugation and characterization of E-[c(RGDfK)_2_] peptides with DSPE-PEG_2000_-COOH

DSPE-PEG_2000_-E-[c(RGDfK)_2_] was synthesized by combining the E-[c(RGDfK)_2_] to the DSPE-PEG_2000_-COOH using the agents EDC and NHS as activators and protectors in a carbodiimide reaction. MALDI–TOF–MS detected the characteristic graphs (Additional file 1: Figure S1a–c) of the two raw materials and synthetic products, confirming the successful binding..

In Additional file 1: Figure S1a–c, the m/z of E-[c(RGDfK)_2_] is 1318.69 (Additional file 1: Figure S1a). Since PEG_2000_ is a polymer with a mass between 1800 and 2200, it shows a flat peak belong to DSPE-PEG_2000_-COOH at about a mass of 2800, as shown in Additional file 1: Figure S1b. The mass of the target product DSPE-PEG_2000_-E-[c(RGDfK)_2_] is around 4100, as showing in Additional file 1: Figure S1c. After comparing the above MALDI–TOF–MS maps, it can be considered that almost all the raw materials are successfully integrated to synthesize DSPE-PEG2000-E-[c(RGDfK)_2_]. The synthesized compound can be used for the targeted modification of NLCs together with the commercial product DSPE-PEG_2000_-FA.

### Particle size, zeta potential and morphology

**Fig. 1:**
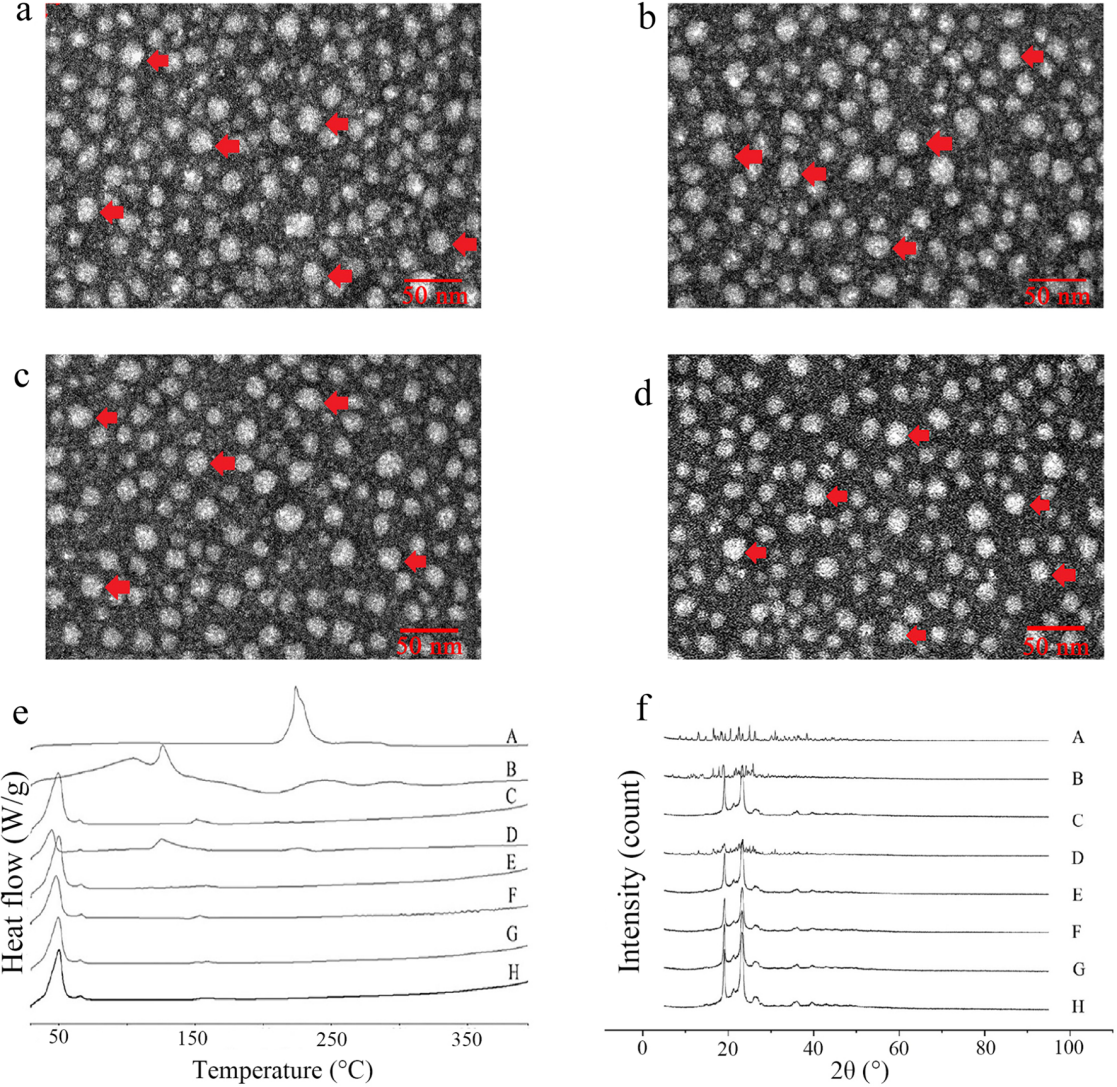
The morphological characteristics of NLC-Sal A/DOX (**a**), FA-NLC-Sal A/DOX (**b**), E-[c(RGDfK)_2_]-NLC-Sal A/DOX (**c**) and E-[c(RGDfK)_2_]/FA-NLC-Sal A/DOX (**d**); DSC results of different formations (**e**); XRD patterns of different formations (**f**). A: DOX reference substance, B: Sal A reference substance, C: Blank-NLC, D: mixture of A-C (w/w, A:B:C = 1:1:1), E: NLC-Sal A/DOX, F: FA-NLC-Sal A/DOX, G: E-[c(RGDfK)_2_]-NLC-Sal A/DOX, H: E-[c(RGDfK)_2_]/FA-NLC-Sal A/DOX

As shown in Table 1, the average particle sizes of DOX/Sal A-NLC, DOX/Sal A-NLC-FA, DOX/Sal A-NLC-E-[c(RGDfK)_2_], and DOX/Sal A-NLC-E-[c(RGDfK)_2_]/FA were about 17–18 nm, the zeta potential varied from − 1.32 to − 4.82 mV, and the PDI was limited between 0.2 and 0.3. These results demonstrated that the prepared NLCs were suitable for delivering DOX and Sal A by taking advantage of enhanced permeability and retention effect. The morphological characteristics of the developed NLCs were observed using a transmission electron microscope (TEM). All tumor-targeted or non-targeted modified NLCs were spherical with rough surfaces (Fig. 1a–d).

**Table 1:** Particle size, PDI, zeta potential and EE of (A) NLC-Sal A/DOX, (B) FA-NLC-Sal A/DOX, (C) E-[c(RGDfK)_2_]-NLC-Sal A/DOX and (D) E-[c(RGDfK)_2_]/FA-NLC-Sal A/DOX

Items	A	B	C	D
Particle size (nm)	18.35 ± 0.21	17.23 ± 0.28	17.39 ± 0.36	18.15 ± 0.23
PDI	0.263 ± 0.035	0.209 ± 0.056	0.203 ± 0.035	0.212 ± 0.065
Zeta (mV)	− 4.32 ± 2.47	− 4.17 ± 1.83	− 4.82 ± 2.74	− 1.32 ± 1.26
Sal A				
EE (%)	82.83 ± 10.59	80.34 ± 6.24	81.87 ± 10.63	80.13 ± 8.59
DOX				
EE (%)	84.87 ± 8.68	79.96 ± 4.26	80.84 ± 7.94	80.36 ± 6.21

Results are expressed as mean ± SD, n = 3

### Differential scanning calorimetry (DSC)

Sal A, DOX, blank-NLC, physical mixture (Sal A: DOX: blank-NLC = 1:1:1), NLC-Sal A/DOX, FA-NLC-Sal A/DOX, FA-NLC-Sal A/DOX, E-[c(RGDfK)_2_]-NLC-Sal A/DOX, and E-[c(RGDfK)_2_]/FA-NLC-Sal A/DOX were characterized by DSC. The melting peaks of Sal A and DOX, as shown in Fig. 1e, were 120 °C and 230 °C, respectively. The melting peaks of the blank-NLC were approximately 60 °C and 150 °C. The melting peaks of the physical mixture (Sal A:DOX:blank-NLC = 1:1:1) were approximately 60 °C, 120 °C, 150 °C, and 230 °C. There was no melting peak for Sal A or DOX in the NLCs, only the peaks for blank-NLC, indicating that the Sal A and DOX were successfully encapsulated in the carriers in amorphous forms.

### X-ray diffraction (XRD)

Sal A, DOX, Blank-NLC, physical mixture (Sal A:DOX:Blank-NLC = 1:1:1), NLC-Sal A/DOX, FA-NLC-Sal A/DOX, FA-NLC-Sal A/DOX, E-[c(RGDfK)_2_]-NLC-Sal A/DOX and E-[c(RGDfK)_2_]/FA-NLC-Sal A/DOX were characterized by XRD. The XRD patterns of DOX and Sal A, as shown in Fig. 1f, showed peaks at 2θ of 13°–30° and 10°–26°, respectively. Furthermore, the blank-NLC has distinct characteristic peaks at 2θ of 19°, 23°, 27°, and 36°. The peaks at 2θ of 13°–30°, 10°–26°,19°, 23°, 27°, and 36° can be found in the physical mixture (Fig. 1f D). Although there are no characteristic peaks of Sal A or DOX in NLCs, the peaks of NLCs are visible. The results show that DOX and Sal A are successfully encapsulated in NLC.

### The drug encapsulation efficiency

The encapsulation efficiency (EE%) of DOX and Sal A in NLCs was evaluated with an HPLC connected to a dual-wavelength ultraviolet detector. Additional file 1: Figure S2 shows the HPLC chromatograms of DOX solution at wavelength 254 nm (Additional file 1: Figure S2a), Sal A solution at wavelength 285 nm (Additional file 1: Figure S2b), the mixture of DOX and Sal A solution at wavelength 254 nm (Additional file 1: Figure S2c), and the mixture of DOX and Sal A solution at wavelength 285 nm (Additional file 1: Figure S2d). The chromatograms indicate that DOX and Sal A have a good resolution. Table 1 shows that EE% of DOX and Sal A were above 79.96% and 82.83%, respectively. The failure to reach almost full-encapsulation may be caused by the fact that both DOX and Sal A are water-soluble ingredients. There was no noticeable difference in EE of DOX and Sal A between the tumor-target modified NLC and unmodified ones, indicating that surface modification did not affect the EE of the DOX and Sal A encapsulated in NLCs in this study.

### In-vitro cellular uptake studies in tumor cells

High-content imaging system was used to measure the fluorescence intensity (FI) of C6 in cells to evaluate the absorption efficiency of the NLCs. Obviously, the FI of all NLC groups was higher than the solution group, which indicates that the cell uptake of C6 in the NLC is faster than C6 in the free form. Generally, the above result may be due to the NLCs usually being taken up by living cells through several endocytic pathways [53]. Meanwhile, the active targeting effect mediated by active targeting substances further promotes the accurate and efficient entry of nanoparticles into corresponding target cells [54].

**Fig. 2:**
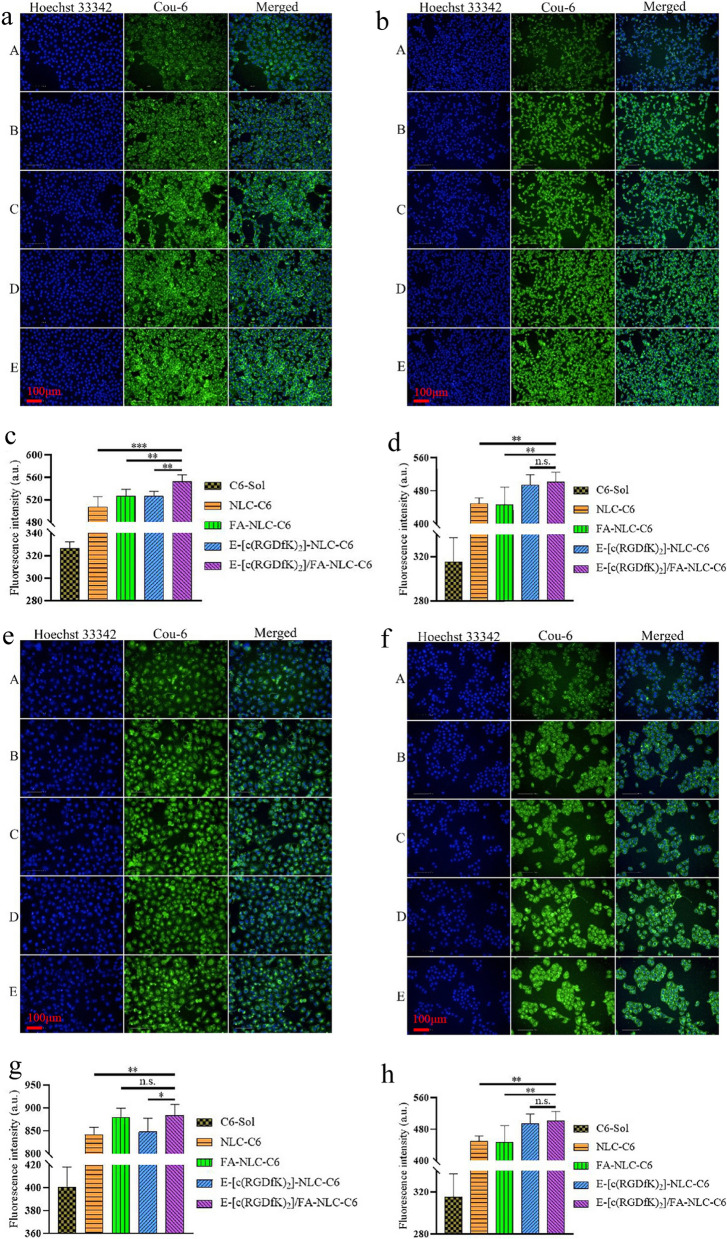
Fluorescent photos and the bar graphs of fluorescence intensity of four tumor cells treated with different preparations after 8 h. Fluorescence picture (**a**) and intensity (**c**) of 4T1 cells, fluorescence picture (**b**) and intensity (**d**) of MDA-MB-231 cells, fluorescence picture (**e**) and intensity (**g**) of MCF-7 cells, fluorescence picture (**f**) and intensity (**h**) of A549 cells. A: C6-Sol, B: NLC-C6, C: FA-NLC-C6, D: E-[c(RGDfK)_2_]-NLC-C6, E: E-[c(RGDfK)_2_]/FA-NLC-C6. *p < 0.05, **p < 0.01. Results are expressed as mean ± SD, n = 3

The results showed that the FI of C6 in cells in all groups increased as the incubation time increased. In the 4T1 cell group (Fig. 2a and c) and MDA-MB-231 cell group (Fig. 2b and d) at 8 h after treatment, the FI of the tumor-targeted modified NLC groups were all greater than the FI of the unmodified NLC group. Using various formulae, the FI of the E-[c(RGDfK)_2_]/FA-Cou-6-NLC group was stronger than the FI of the two single tumor-homing target modification groups. These results indicate that both the cell lines have FR and integrin α_v_β_3_ receptor on the cell surface, as verified by the cell uptake test after the receptor saturated treatment (Additional file 1: Figure S3a–d).

In MCF-7 cells, at 8 h (Fig. 2e and g), the FI of the two NLCs groups with FA as the targeting modifier was greater than the FI of other NLC groups, which is due to the MCF-7 cell line expressing FR but not expressing the integrin α_v_β_3_ receptor (verified by the cell pretreatment saturated experiment shown in Additional file 1: Figure S3e and g). In contrast, in A549 cells at 8 h (Fig. 2f and h), the FI of the two NLCs groups with E-[c(RGDfK)_2_] as the targeting modifier was greater than the FI of other NLC groups, which is due to the A549 cell line expressing the integrin α_v_β_3_ receptor but not expressing FR (verified by the cell pretreatment saturated experiment shown in Additional file 1: Figure S3f and h).

Overall, by modifying the NLC with E-[c(RGDfK)_2_]/FA, the cellular intake by tumor cells with the FR and/or the integrin α_v_β_3_ receptor on the surface can be enhanced, which reflects the active tumor-targeting effect of the E-[c(RGDfK)_2_]/FA-NLC. Of note, 4T1 and MDA-MB-231 cell lines are triple-negative breast cancer (TNBC) cell lines [55]; thus E-[c(RGDfK)_2_]/FA-NLC maybe be a promising anti-tumor drug delivery carrier for TNBC.

### In-vitro renal cell viability and mitochondrial characterization

**Fig. 3:**
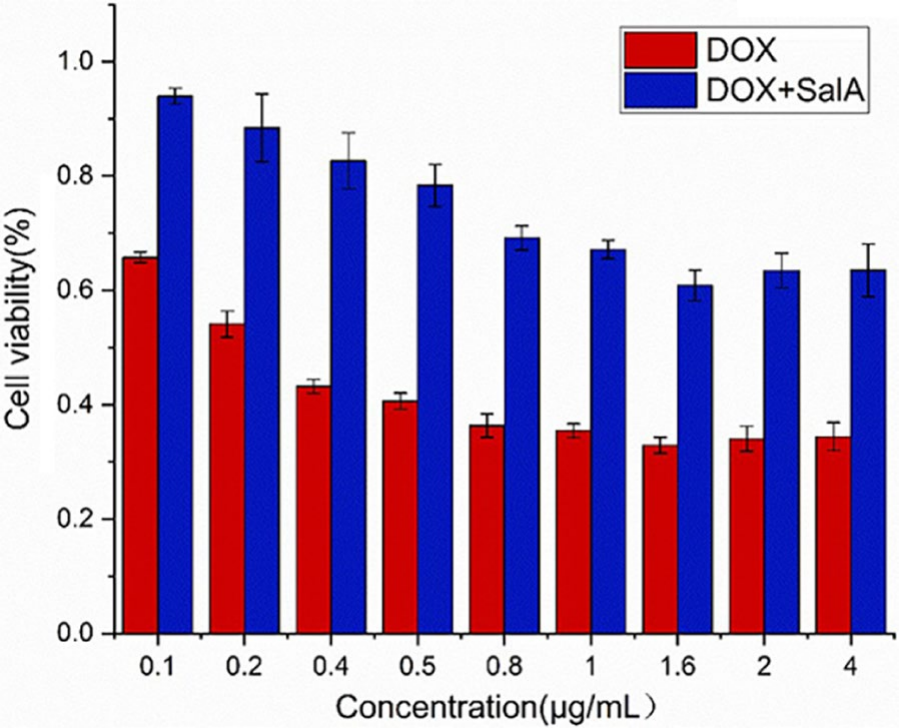
The cell viability of HEK293 cells treated with DOX solution and DOX/Sal A solution. The concentration range of DOX was 0.4–4.0 μg/mL, and the concentration of Sal A at each point was about 10 times to that of DOX

The cell viability was tested using the CCK-8 kit. The cell viability after treatment with DOX solution and DOX/Sal A solution are shown in Fig. 3. The results showed that Sal A could significantly reduce the damage caused by DOX to HEK293 cells when the dosage of Sal A was 10 times that of DOX (0.1–4.0 μg/mL); this implies that Sal A reduces DOX-induced cellular oxidative stress, downregulates the expression of NF-κB p65 and p-IκBα, and simultaneously upregulates the expression of IκBα protein [43].

**Fig. 4:**
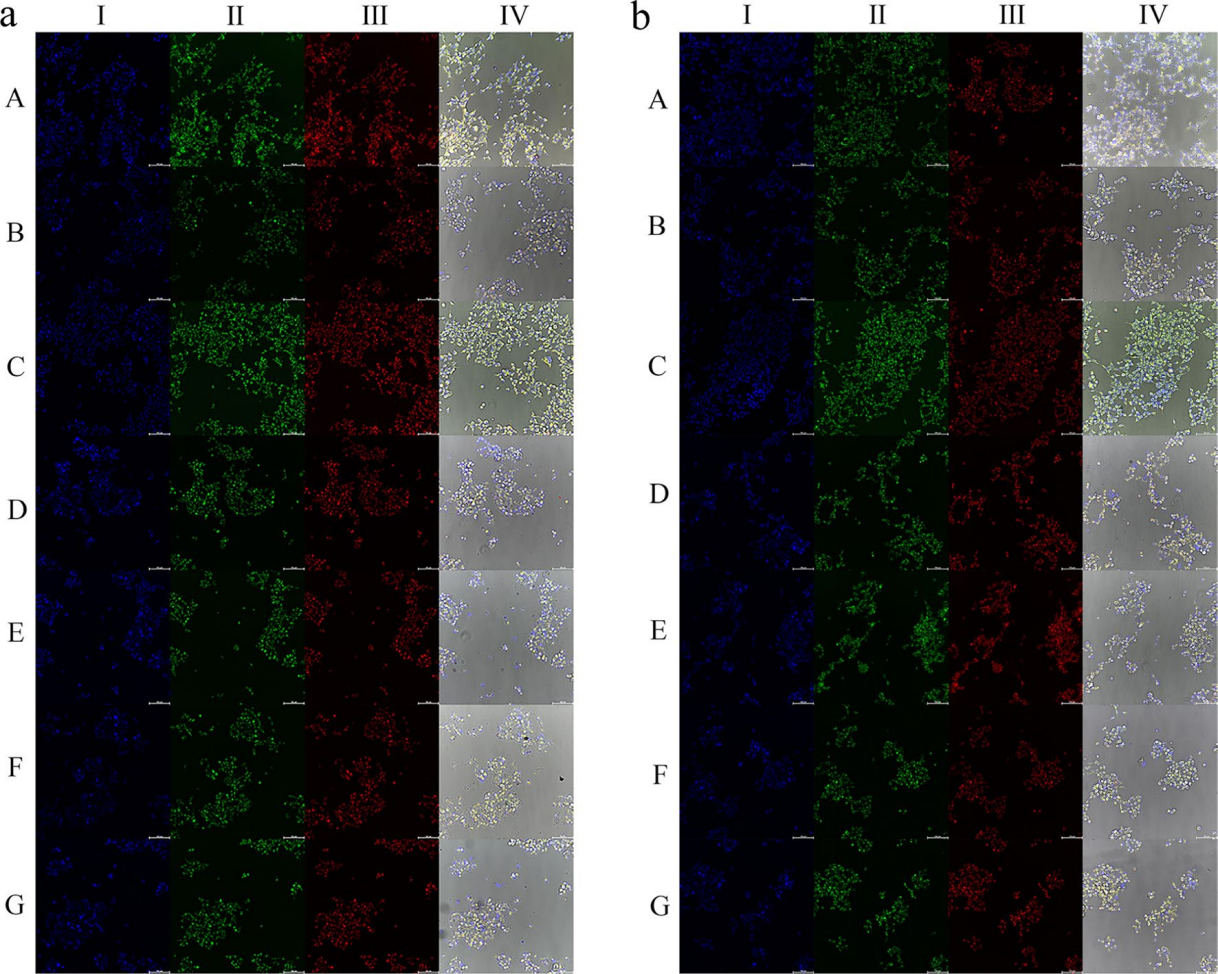
The fluorescence staining images of HEK293 cells treated with the preparations after 4 h (**a**) and 24 h (**b**). A: N.S., B: DOX solution, C: Sal A/DOX solution, D: NLC-Sal A/DOX, E: FA-NLC-Sal A/DOX, F: E-[c(RGDfK)_2_]-NLC-Sal A/DOX, G: E-[c(RGDfK)_2_]/FA-NLC-Sal A/DOX. I: Hoechst 33342, II: Rhodamine 123, III: Mitotracker Deep Red FM, IV: The full view of the cells

**Fig. 5:**
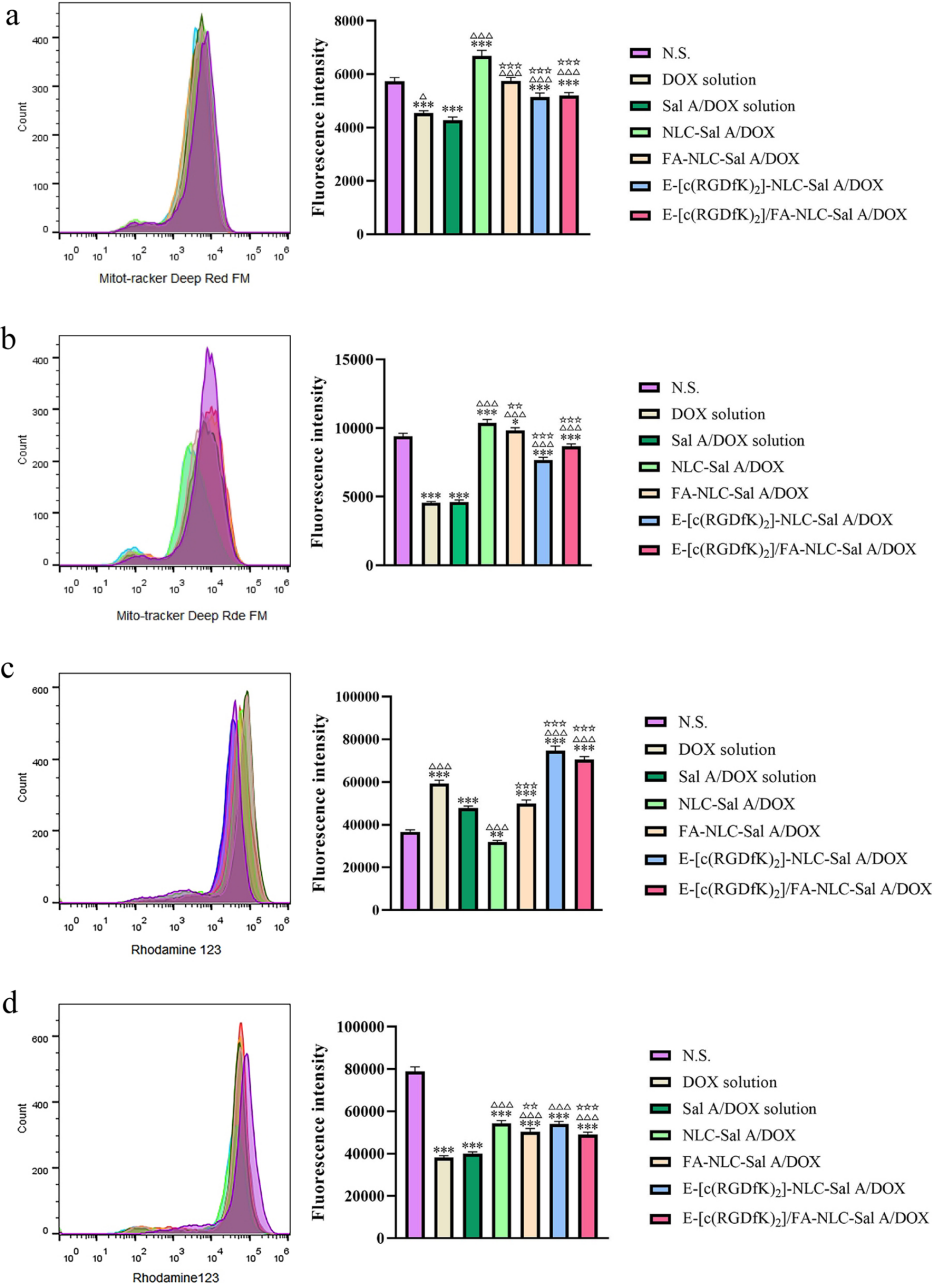
Merged flow cytometric histograms and fluorescence quantitative bar graphs of Mitotracker Deep Red FM in HEK293 cells treated with the preparations after 4 h (**a**) and 24 h (**b**). Merged flow cytometric histograms and fluorescence quantitative bar graphs of Rhodamine 123 in HEK293 cells treated with the preparations after 4 h (**c**) and 24 h (**d**). ***p < 0.001 vs N.S., *p < 0.05 vs N.S., **p < 0.01 vs N.S., ^△^*p* < 0.05 vs DOX/Sal A solution, ^△△△^*p* < 0.001 vs DOX/Sal A solution, ^☆☆^*p* < 0.01 vs NLC-DOX/Sal A, ^☆☆☆^*p* < 0.001 vs NLC-DOX/Sal A. Results are expressed as mean ± SD, n = 3

The fluorescence staining images of HEK293 cells treated with the preparations of 4 h and 24 h were observed by Operetta^®^ high-content imaging system, as shown in Fig. 4. In the fluorescence quantitative analysis of mitotracker Deep Red FM (Fig. 5a and b), we found that after incubation of 4 h and 24 h, both the DOX solution and DOX/Sal A solution groups clearly indicated more change on mitochondrial mass than the NLC related preparations, which indicated that loading the drug into NLC could significantly alleviate the effect of the drug on mitochondrial mass. Significant differences were observed between the fluorescence intensity of NLC-DOX/Sal A and that of three other modified preparations groups, which suggested that the surface modification on NLC also altered the effect of the NLC preparation on mitochondrial mass in renal cells.

In the fluorescence quantitative analysis of rhodamine 123 (Fig. 5c and d), the mitochondrial membrane potential of renal cells in each group after 4 h and 24 h of incubation were compared, and the mitochondrial membrane potential in DOX solution and DOX/Sal A solution groups decreased more than the NLC related preparations, which demonstrated that loading the DOX in NLC could considerably diminish the effect of the DOX on the mitochondrial membrane potential. Changes in mitochondrial mass and membrane potential were intimately associated to the occurrence of apoptotic cascade [56, 57]. Therefore, it was useful to limit DOX-induced renal cytotoxicity by loading DOX into NLC preparations to reduce DOX-induced abnormalities in mitochondrial mass and membrane potential. However, after 24 h, the fluorescence intensity of NLC-DOX/Sal A was significantly stronger than that of FA-NLC-Sal A/DOX and E-[c(RGDfK)_2_]/FA-NLC-Sal A/DOX, which indicated the toxicity of NLC preparations to renal cell mitochondria was affected by different modifications on the surface of NLC.

### In-vivo bio-distribution

**Fig. 6:**
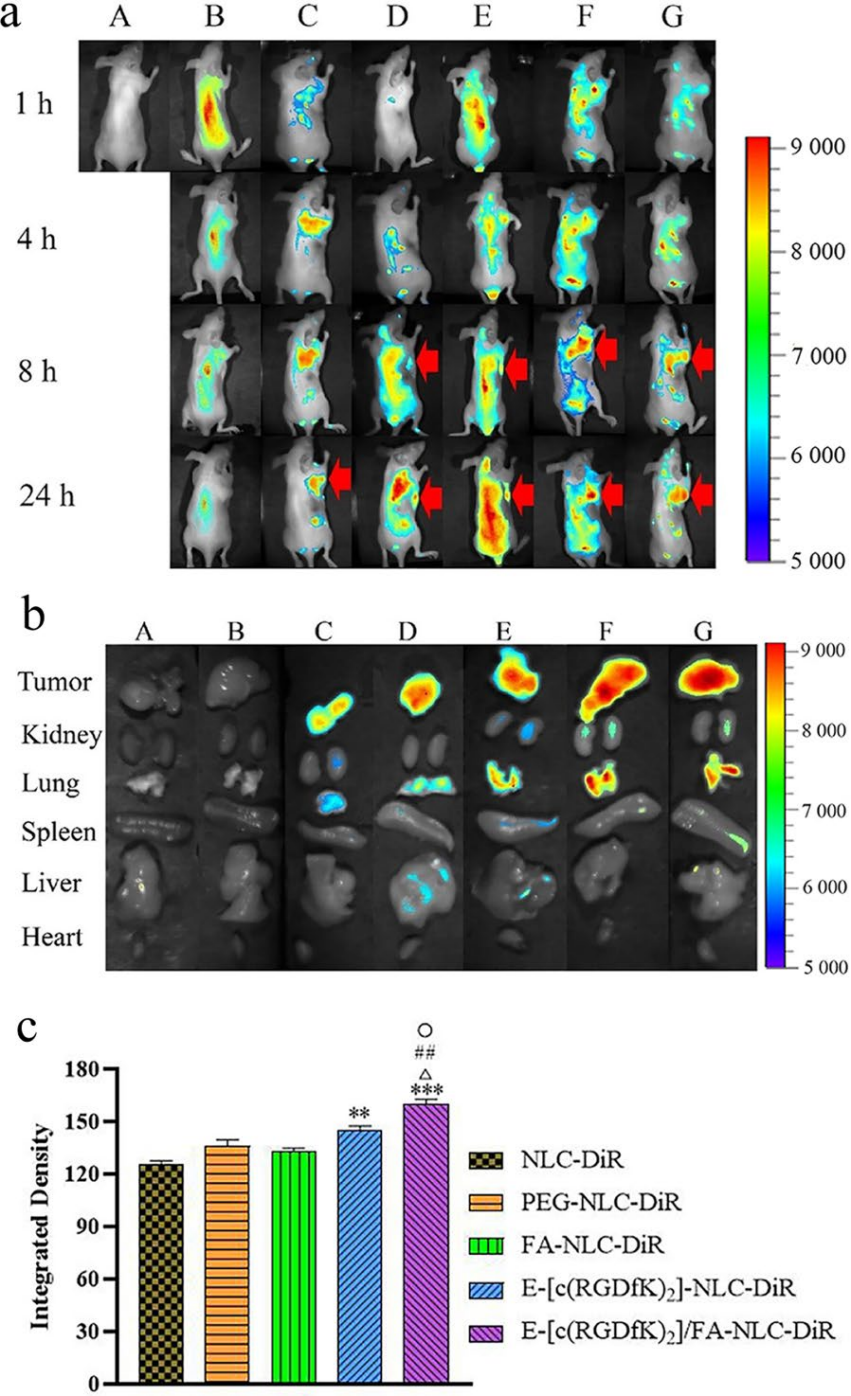
**a** The bio-distribution of the preparations in tumor-bearing female BALB/c nude mice, **b** Fluorescence images of the organs and tumor tissues of the mice treated with different preparations at different times, **c** FI statistical data of the tumor tissues excised from the mice after being treated with different formulations at 24 h. A: Normal saline, B: DiR solution, C: NLC-DiR, D: PEG-NLC-DiR, E: FA-NLC-DiR, F: E-[c(RGDfK)_2_]-NLC-DiR, G: E-[c(RGDfK)_2_]/FA-NLC-DiR. ***p < 0.001 vs NLC-DiR, **p < 0.01 vs NLC-DiR, ^△^*p* < 0.05 vs PEG-NLC-DiR, ^○^*p* < 0.05 vs E-[c(RGDfK)_2_]-NLC-DiR, ^##^*p* < 0.01 vs FA-NLC-DiR. Results are expressed as mean ± SD, n = 3

Figure 6a shows that free DiR did not accumulate at the tumor site, owing to rapid clearance and non-specific distribution after intravenous injection. There was no DiR accumulation in tumor tissues in any group during the first 4 h. However, after 8 h, visible FI was seen in the tumor site in the NLC groups. The tumor-targeting distribution outcomes resulted from the systemic circulation characteristics conferred by the PEG linked on the surface of the NLCs [58], the effects of enhanced permeability and retention (EPR) in the cancer tissues, and the active targeting endowed by E-[c(RGDfK)_2_] and/or FA modified on the surface of the NLCs. These results also show that the FI at the tumor site was the highest level at 24 h, and the FI of the E-[c(RGDfK)_2_]/FA-DOX/Sal A-NLC group was the strongest among all the groups. This may be due to the high-affinity between E-[c(RGDfK)_2_]/FA and receptors on the surface of 4T1 cells, which mediated the internalization of the NLCs into the cell [36, 59]. The in vitro DiR distribution and accumulation in organs and tumors is shown in Fig. 6b. It was certain that no FI was present in the organs or tumors in the DiR solution group. Figure 6c showed that the E-[c(RGDfK)_2_]/FA-NLC-DiR group possessed significantly stronger FI than the single modification groups. It is worth noting that fluorescence was observed in the lungs of the five NLC groups, resulting from the deposition of the NLC in the dense blood vessels in the lungs [60].

### In-vivo study of creatinine (Cre) concentration, alanine aminotransferase (ALT) activity and creatine kinase (CK) activity

The serum Cre concentration is completely dependent on its excretion rate by the kidney [61]. Therefore, an increase in Cre concentration in vivo often accompanies by renal functional damage. The DOX could result in nephrotic syndrome and hence increase the serum Cre concentration [62].

**Fig. 7:**
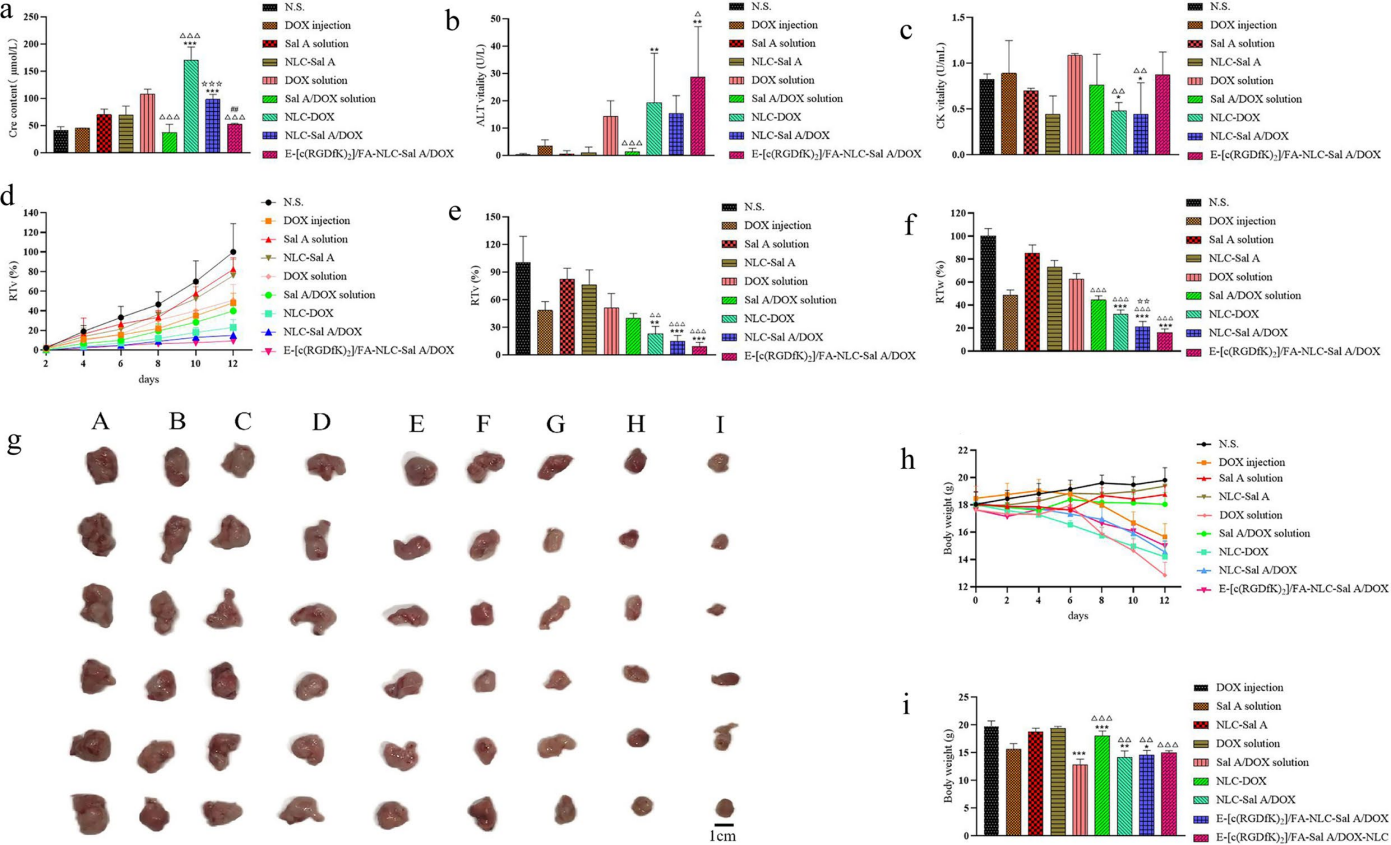
The concentration of Cre (**a**), the vitalities of ALT (**b**) and CK (**c**) in the serum, the RTv of different preparations at the treatment times (**d**), the RTv of different preparations at the end of the treatment (**e**), the RTw of different preparations at the end of the treatment (**f**), the isolated tumor tissues after being treated 12 days with different preparations (**g**), body weights of the tumor-bearing female BALB/c mice treated with different preparations at the treatment times (**h**), the body weights after being treated 12 days (**i**). A: N.S., B: Sal A solution, C: NLC-Sal A, D: DOX solution, E: DOX injection, F: Sal A/DOX solution, G: NLC-DOX, H: NLC-Sal A/DOX, I: E-[c(RGDfK)_2_]/FA-NLC-Sal A/DOX. **p < 0.01 vs DOX injection. *p < 0.05 vs DOX injection, **p < 0.01 vs DOX injection, ***p < 0.001 vs DOX injection, ^△^*p* < 0.05 vs DOX solution, ^△△^*p* < 0.01 vs DOX solution, ^△△△^*p* < 0.001 vs DOX solution, ^☆☆^*p* < 0.01 vs NLC-DOX ^☆☆☆^*p* < 0.001 vs DOX-NLC, ^##^*p* < 0.01 vs Sal A/DOX-NLC. Results are expressed as mean ± SD, n = 6

Actually, as shown in Fig. 7a, the serum Cre concentration in the Sal A/DOX solution group was significantly reduced compared with the DOX solution group (*p* < 0.001). The same level of reduction in serum Cre also appeared in the comparison of Sal A/DOX-NLC group with DOX-NLC (*p* < 0.001). The results show that Sal A could effectively antagonize the renal toxicity of DOX [43]. It is worth noting that the serum Cre concentration in the E-[c(RGDfK)_2_]/FA-Sal A/DOX-NLC group was remarkably lower than that in the Sal A/DOX-NLC group, which implied that the E-[c(RGDfK)_2_]/FA tumor-target modification could significantly reduce the kidney toxicity of the DOX loaded in the NLC.

Increased activity of ALT in serum means liver damage [63]. As shown in Fig. 7b, in the groups containing DOX, the ALT in serum was significantly increased compared with the control group, demonstrating the liver toxicity of the DOX [64]. Compared with the DOX solution group, the ALT activity in serum of the E-[c(RGDfK)_2_]/FA-Sal A/DOX-NLC group obviously increased. It might be caused by the absorption of FA in the liver [65], which resulted in the accumulation of DOX and Sal A loaded in FA modified NLC in the liver. It indicated that the FA-modified drug delivery system would target not only the tumor but also the liver, which requested researchers to focus on liver toxicity while using FA-modified carriers.

The activity of serum CK increases when muscle cells are damaged. As shown in Fig. 7c, the activity of CK was measured to evaluate the toxicity of the formulations to muscle cells. The results showed that the muscle cell damage in the DOX solution group was more serious than that in the NLC-DOX group (*p* < 0.01), suggesting that NLC could reduce the damage of DOX to muscle cells. Compared with NLC-Sal A/DOX, the activity of CK in E-[c(RGDfK)_2_]/FA-NLC-Sal A/DOX group increased slightly without significance, and the mechanism needs further experimental verification.

### In-vivo antitumor activity study

By measuring and recording the change in tumor volume after administration of preparations, the isolated tumor weight, the RTv, RTw, IRv, and IRw were calculated to evaluate the inhibitory potential of the preparations on the 4T1 cells. As shown in Fig. 7d–g, compared with the N.S. group, the DOX solution group and the DOX injection group both exhibited a significant inhibitory effect on RTv and RTw. In contrast, Sal A solution group and NLC-Sal A group did not show evident inhibitory effects. Compared with the DOX solution group, NLC-Sal A/DOX (*p* < 0.001) and E-[c(RGDfK)_2_]/FA-NLC-Sal A/DOX (*p* < 0.001) groups showed a significant reduction in RTv and RTw. Among the nine treated groups, the E-[c(RGDfK)_2_]/FA-NLC-Sal A/DOX group showed the best inhibitory effect as measured by tumor volume and weight. The IRv and IRw of E-[c(RGDfK)_2_]/FA-NLC-Sal A/DOX were the most prominent among the nine treated groups, as shown in Additional file 2: Table S1 (90.72% and 83.94%, respectively).

The weight change of tumor-bearing mice during the treatment is one of the safety monitoring indicators of anti-tumor drugs. As shown in Fig. 7h and i, the body weights of Sal A solution, NLC-Sal A and Sal A/DOX solution maintained the same level as the N.S. group. The results show that Sal A will not affect body weight as DOX, and it also can antagonize the weight loss caused by DOX in the form of solution. Obviously, the DOX solution caused a drastic reduction in body weight (*p* < 0.001, vs N.S.).

The E-[c(RGDfK)_2_]/FA-NLC-Sal A/DOX revealed a similar degree of toxicity with the DOX injection, and its Ocs of heart, lung, and kidney have no significant difference vs N.S. (Additional file 1: Figure S4a–c), respectively. The enlargement of the liver in E-[c(RGDfK)_2_]/FA-NLC-Sal A/DOX group may be related to the liver cell damage caused by the phagocytosis of the drug-loaded NLCs by the reticuloendothelial cell phagocytic system [66]. The Ocs of the spleen in the N.S., Sal A solution and NLC-Sal A groups were swelling, which was due to the splenomegaly induced by the inoculation of 4T1 cells into the mammary fat pad of female BALB⁄c mice [67]. At the same time, the DOX reduced splenomegaly in addition to blockade of tumor growth [68].

### Histological examination

**Fig. 8:**
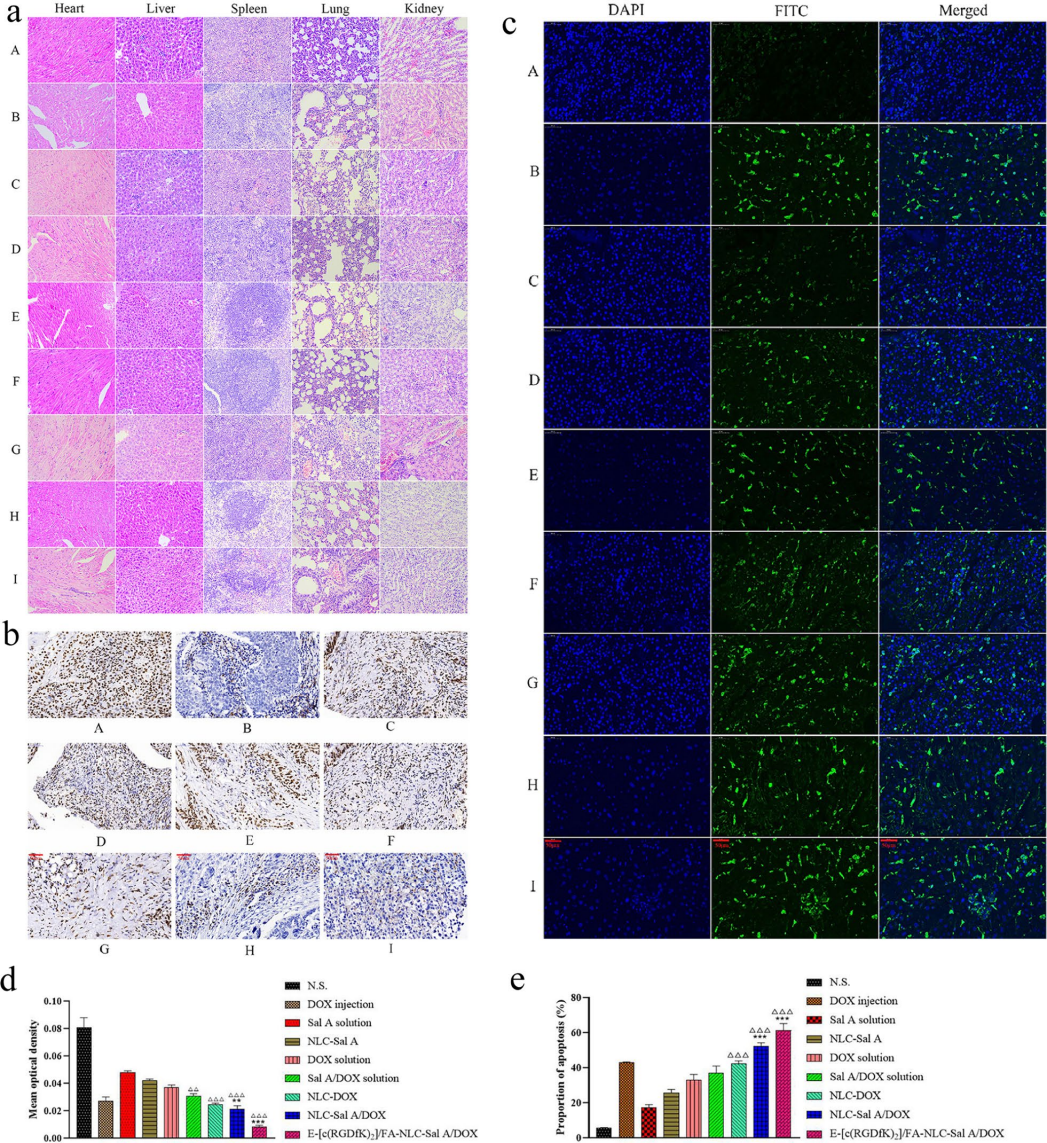
HE staining of the organs (**a**), Ki67 staining (**b**), proportion of proliferation (**d**), TUNEL staining (**c**) and proportion of apoptosis (**e**) of the tumor tissues obtained from the tumor-bearing female BALB/c mice treated with different preparations. All the pictures were gained under the magnification of 400×. Scale bar = 50 μm. A: N.S., B: Sal A solution, C: NLC-Sal A, D: DOX solution, E: DOX injection, F: Sal A/DOX solution, G: NLC-DOX, H: NLC-Sal A/DOX, I: E-[c(RGDfK)_2_]/FA-NLC-Sal A/DOX. **p < 0.01 vs DOX injection, ***p < 0.001 vs DOX injection, ^△△^*p* < 0.01 vs DOX solution, ^△△△^*p* < 0.001 vs DOX solution. Results are expressed as mean ± SD, n = 6

The results of HE (Fig. 8a) indicate that DOX does significant damage to the tissues of the heart, liver, lung, kidney, which is caused by the DOX-induced formation of free radicals and the injury to DNA [69]. Meanwhile, the capture of the NLC by the reticuloendothelial system aggravates the cumulative damage to the liver caused by the DOX loaded in the carrier [67]. The deposition and concentration of DOX and NLC in the lungs due to the dense capillaries, was the main cause of lung injury in this research [70]. The HE results of DOX injection, DOX solution, and NLC-DOX groups suggested renal damage. In the solution and the NLC forms, it was found that with the inclusion of Sal A, the injury caused by DOX to the kidney was significantly reduced, which is related to the antioxidant properties of Sal A [43].

### Immunohistochemistry analysis

The result of Ki67 staining was used to assess the proliferation of tumor cells in vivo. It can be seen from Fig. 8b and d that the Ki67 expression level of E-[c(RGDfK)_2_]/FA-NLC-Sal A/DOX is significantly lower than DOX solution (*p* < 0.001), indicating that E-[c(RGDfK)_2_]/FA functionalized NLC-Sal A/DOX possess good antiproliferative activity against 4T1 cell. Additionally, the TUNEL test (Fig. 8c and e) was used to detect apoptosis in tumor tissues. The results showed that the proportion of positive nuclei (green) increased in the E-[c(RGDfK)_2_]/FA-NLC-Sal A/DOX group compared with the NLC-Sal A/DOX group, indicating that NLC-Sal A/DOX modified with E-[c(RGDfK)_2_]/FA could deliver the drug into cells more efficiently than the unfunctionalized preparation to induce more 4T1 cells apoptosis.

## Conclusion

In this study, DOX and Sal A were co-loaded in NLC modified by E-[c(RGDfK)_2_]/FA to prepare E-[c(RGDfK)_2_]/FA-NLC-Sal A/DOX. The synthesized multifunctional nano-drug-delivery carrier possesses the characteristics of small size and high encapsulation efficiency. This preparation could enhance the anti-tumor effect of Sal A/DOX in the 4T1 mouse tumor model, increase apoptosis of tumor cells, and decrease proliferation rate of tumor cells. The Sal A in the formulation successfully antagonized the nephrotoxicity of DOX in vivo. Meanwhile, the double-targeted modification of E-[c(RGDfK)_2_]/FA also significantly reduced the damage to the kidneys induced by DOX. Therefore, we believe that the E-[c(RGDfK)_2_]/FA-NLC-Sal A/DOX, as a multidrug delivery carrier based on nanoparticles, can be a potential new targeted therapeutic strategy in various types of tumors, especially breast cancer. Concurrently, this novel drug-delivery system has the potential to greatly alleviates renal toxicity in cancer patients during the treatment process, thereby improving patient tolerance to anti-tumor therapy.

## Supplementary Information

**Additional file 1: Figure S1.** MALDI–TOF mass spectra of E-[c(RGDfK)_2_] (a), DSPE-PEG_2000_-COOH (b) and DSPE-PEG_2000_-E-[c(RGDfK)_2_] (c). **Figure S2.** HPLC chromatograms of DOX solution at wavelength 254 nm (a), Sal A solution at wavelength 285 nm (b), the mixture of DOX/Sal A solution at wavelength 254 nm (c), and the mixture of DOX/Sal A solution at wavelength 285 nm (d). Peak 1 is DOX, and peak 2 is Sal A. **Figure S3.** Fluorescent photos of four tumor cells in the cell uptake test after the receptor saturated treatment. Fluorescence picture (a) and intensity (c) of 4T1 cells, fluorescence picture (b) and intensity (d) of MDA-MB-231 cells, fluorescence picture (e) and intensity (g) of MCF-7 cells, fluorescence picture (f) and intensity (h) of A549 cells. A: FA+/FA-NLC-C6, B: FA-NLC-C6, C: E-[c(RGDfK)_2_]+/E-[c(RGDfK)_2_]-NLC-C6, D: E-[c(RGDfK)_2_]-NLC-C6. **p < 0.01. Results are expressed as mean ± SD, n = 3. **Figure S4.** The Ocs of the isolated heart (a), lung (b), kidney (c), liver (d) and spleen (e) from the tumor-bearing female BALB/c mice after being treated with different preparations 12 days. *p < 0.05 vs DOX injection, **p < 0.01 vs DOX injection, ^△△^*p* < 0.01 vs DOX solution. Results are expressed as mean ± SD, n = 6.

**Additional file 2: Table S1.** The IRV and IRw of the isolated tumor tissues excised from the tumor-bearing female BALB/c mice treated with different preparations.

## Data Availability

The datasets used and/or analyzed during the current study are available from the corresponding author on reasonable request.

## Abbreviations

CCK-8: Cell Counting Kit-8
Cou-6: Coumarin 6
DOX: Doxorubicin hydrochloride
Sal A: Salvianolic acid A
DiR: 1,10-Dioctadecyl-3,3,3,3-tetramethyl indotricarbocyanine iodide
DSPE-PEG_2000_: 1,2-Distearoyl-sn-glycero-3-phospho ethanolamine-*N*-[methoxy(polyethyl ene glycol)-2000]
DMSO: Dimethyl sulfoxide
DSC: Differential scanning calorimetry
E-[c(RGDfK)_2_]: Glu- Cyclo (Arg-Gly-Asp-Phe-Lys)_2_
EDC: 1-Ethyl-3-(3-dimethylaminopropyl) carbodiimide
EE: Encapsulation efficiency
FI: Fluorescence intensity
FA: Folate acid
HE: Histological examination
IRv: Tumor volume inhibition rate
IRw: Tumor weight inhibition rate
IVIS: In-vivo imaging system
Miglyol ® 812: Caprylic/capric triglyceride
Myrj 52: Polyoxyethylene (40) stearate
NHS: *N*-Hydroxysuccinimide
NLC: Nanostructured lipid carriers
PDI: Polydispersity Index
TEM: Transmission electron microscope
XRD: X-ray diffraction
Cre: Creatinine
ALT: Alanine aminotransferase
CK: Creatine kinase
