# Supplementary material for: The anti-tumor and renoprotection study of E-[c(RGDfK)2]/folic acid co-modified nanostructured lipid carrier loaded with doxorubicin hydrochloride/salvianolic acid A
Source: J Nanobiotechnology. 2022 Sep 24;20:425. doi: 10.1186/s12951-022-01628-x (PMC9509648; doi:10.1186/s12951-022-01628-x)
Supplement: Supplementary file 1 — Additional file 1: Figure S1. MALDI–TOF mass spectra of E-[c(RGDfK)2] (a), DSPE-PEG2000-COOH (b) and DSPE-PEG2000-E-[c(RGDfK)2] (c). Figure S2. HPLC chromatograms of DOX solution at wavelength 254 nm (a), Sal A solution at wavelength 285 nm (b), the mixture of DOX/Sal A solution at wavelength 254 nm (c), and the mixture of DOX/Sal A solution at wavelength 285 nm (d). Peak 1 is DOX, and peak 2 is Sal A. Figure S3. Fluorescent photos of four tumor cells in the cell uptake test after the receptor saturated treatment. Fluorescence picture (a) and intensity (c) of 4T1 cells, fluorescence picture (b) and intensity (d) of MDA-MB-231 cells, fluorescence picture (e) and intensity (g) of MCF-7 cells, fluorescence picture (f) and intensity (h) of A549 cells. A: FA+/FA-NLC-C6, B: FA-NLC-C6, C: E-[c(RGDfK)2]+/E-[c(RGDfK)2]-NLC-C6, D: E-[c(RGDfK)2]-NLC-C6. **p < 0.01. Results are expressed as mean ± SD, n = 3. Figure S4. The Ocs of the isolated heart (a), lung (b), kidney (c), liver (d) and spleen (e) from the tumor-bearing female BALB/c mice after being treated with different preparations 12 days. *p < 0.05 vs DOX injection, **p < 0.01 vs DOX injection, △△p < 0.01 vs DOX solution. Results are expressed as mean ± SD, n = 6. [file 12951_2022_1628_MOESM1_ESM.docx]

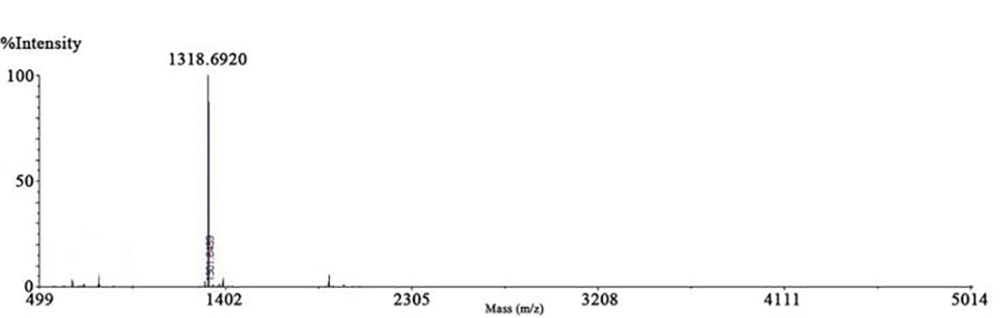


a


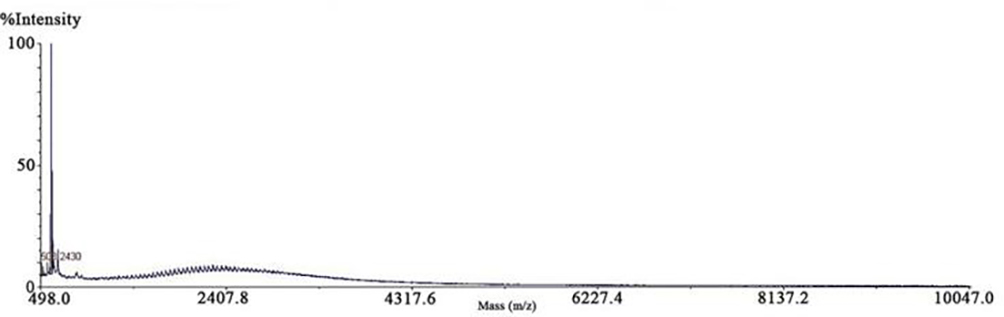


b


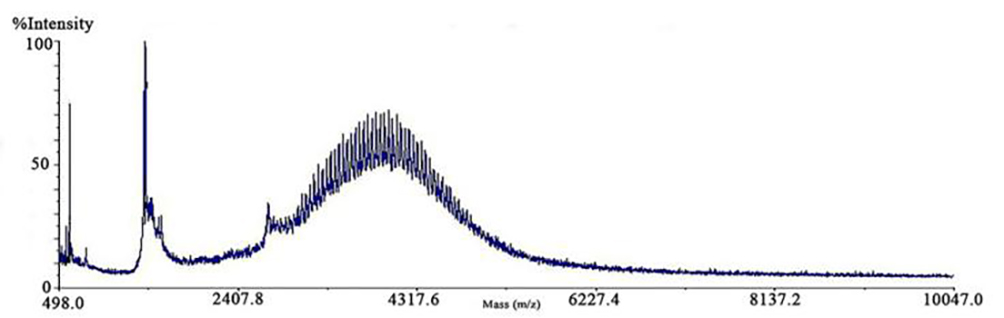


c

Figure S1. MALDI-TOF mass spectra of E-[c(RGDfK)_2_] (a), DSPE-PEG_2000_-COOH (b) and DSPE-PEG_2000_-E-[c(RGDfK)_2_] (c).


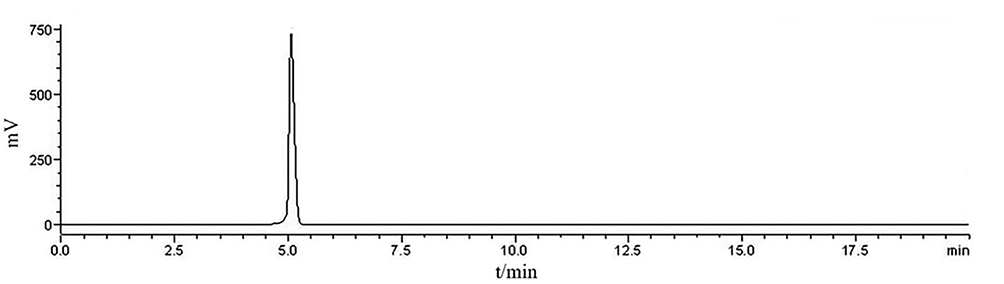


a


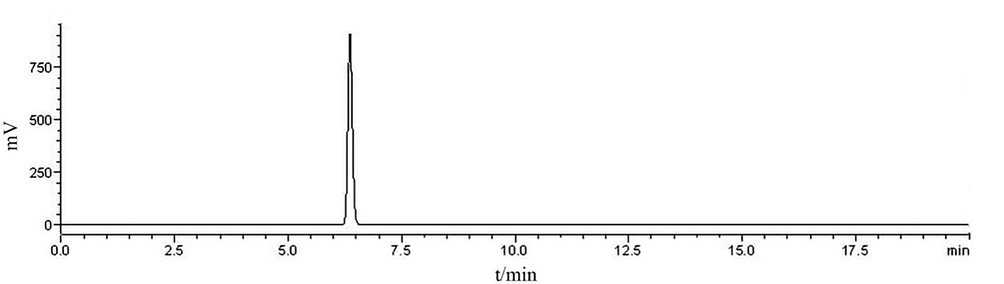


b


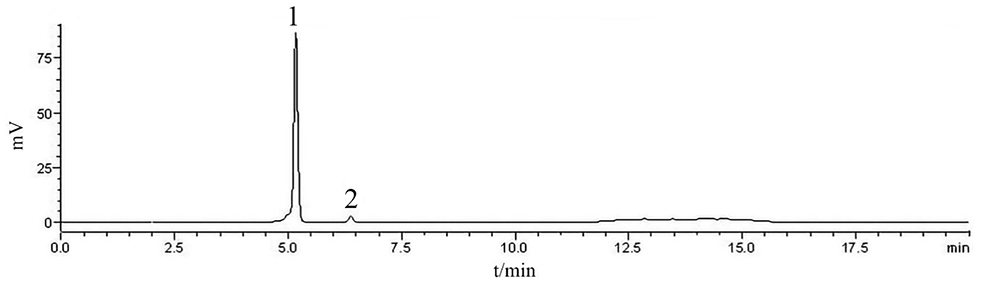


c


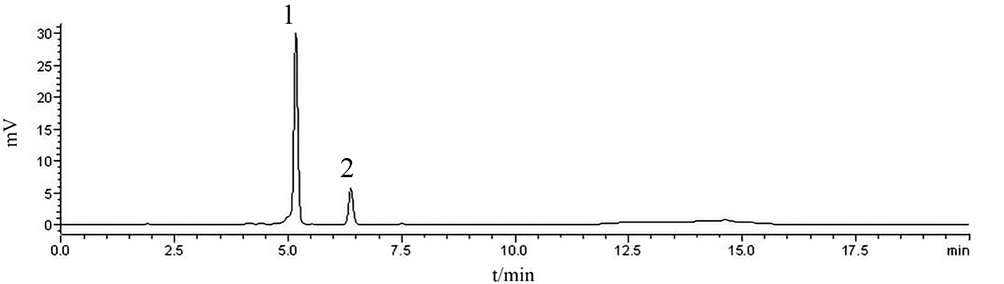


d

Figure S2. HPLC chromatograms of DOX solution at wavelength 254 nm (a), Sal A solution at wavelength 285 nm (b), the mixture of DOX/Sal A solution at wavelength 254 nm (c), and the mixture of DOX/Sal A solution at wavelength 285 nm (d). Peak 1 is DOX, and peak 2 is Sal A.


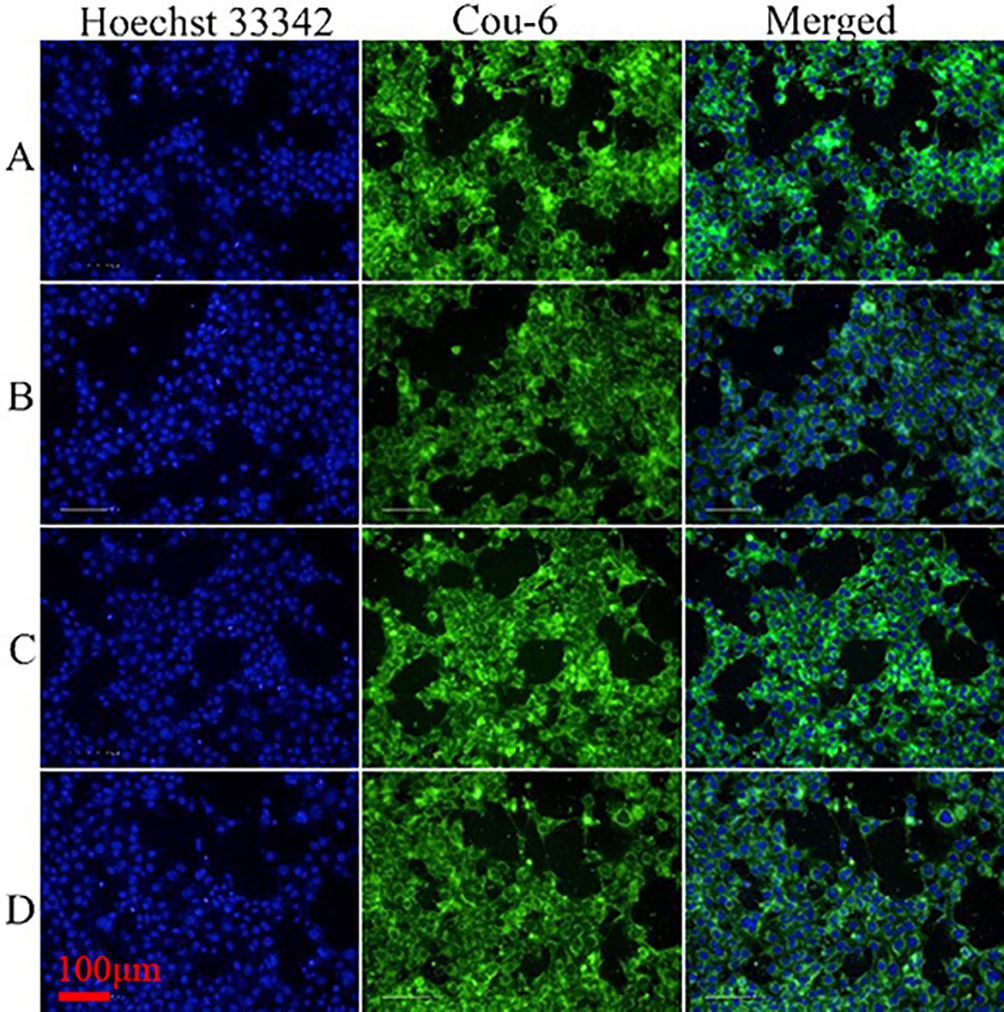


a


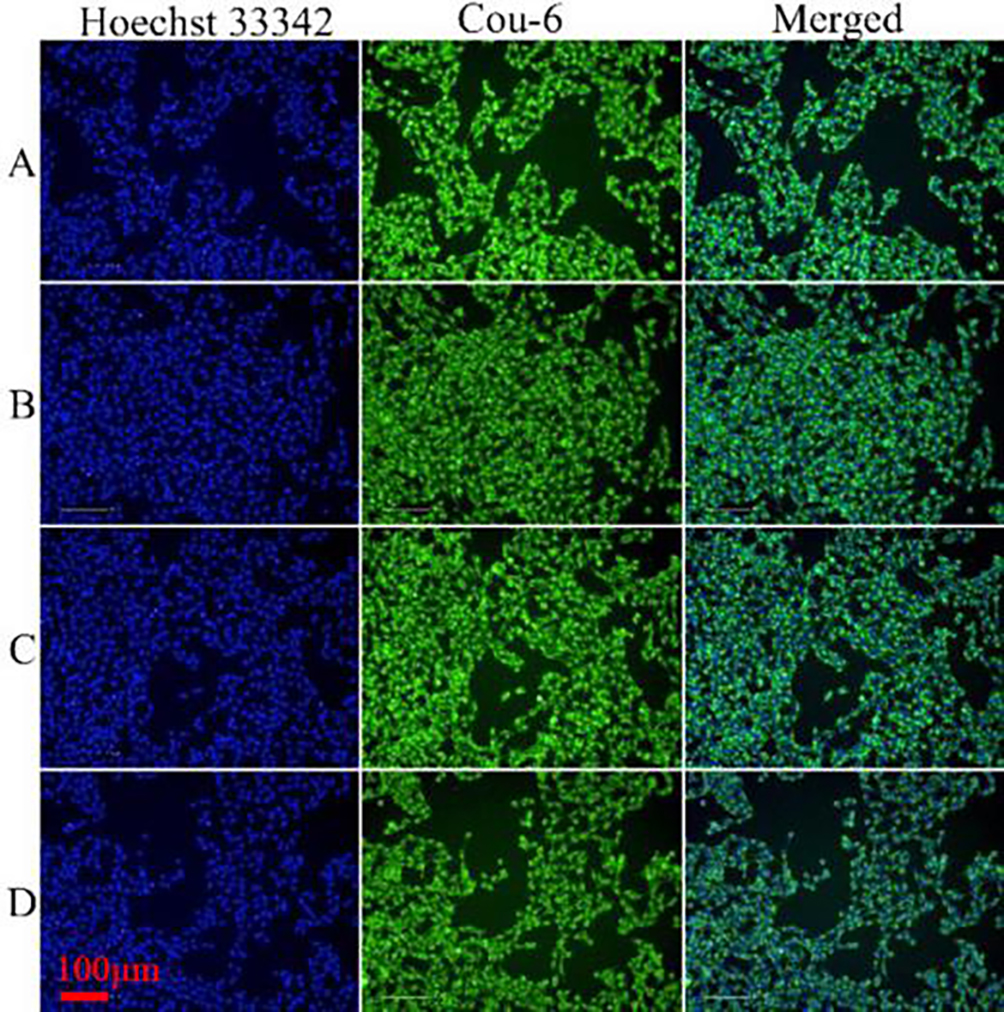


b


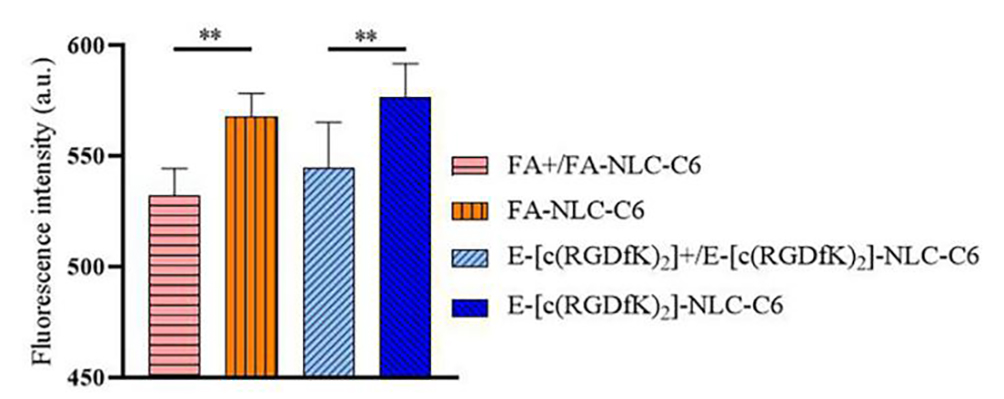


c


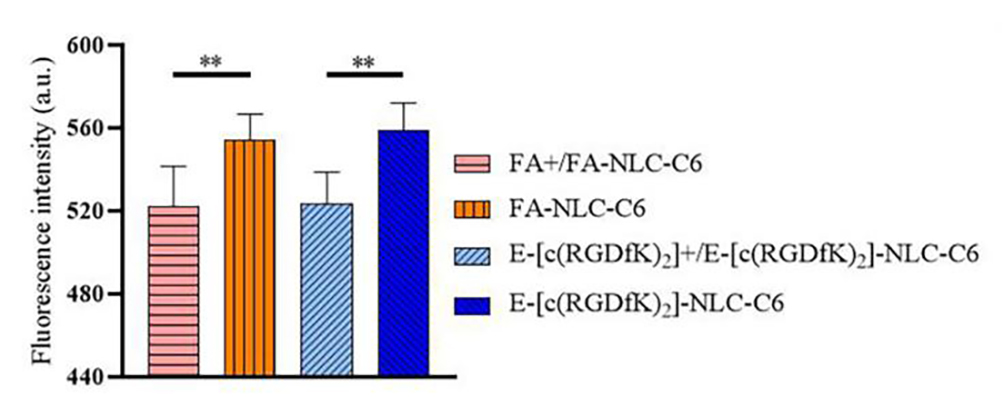


d


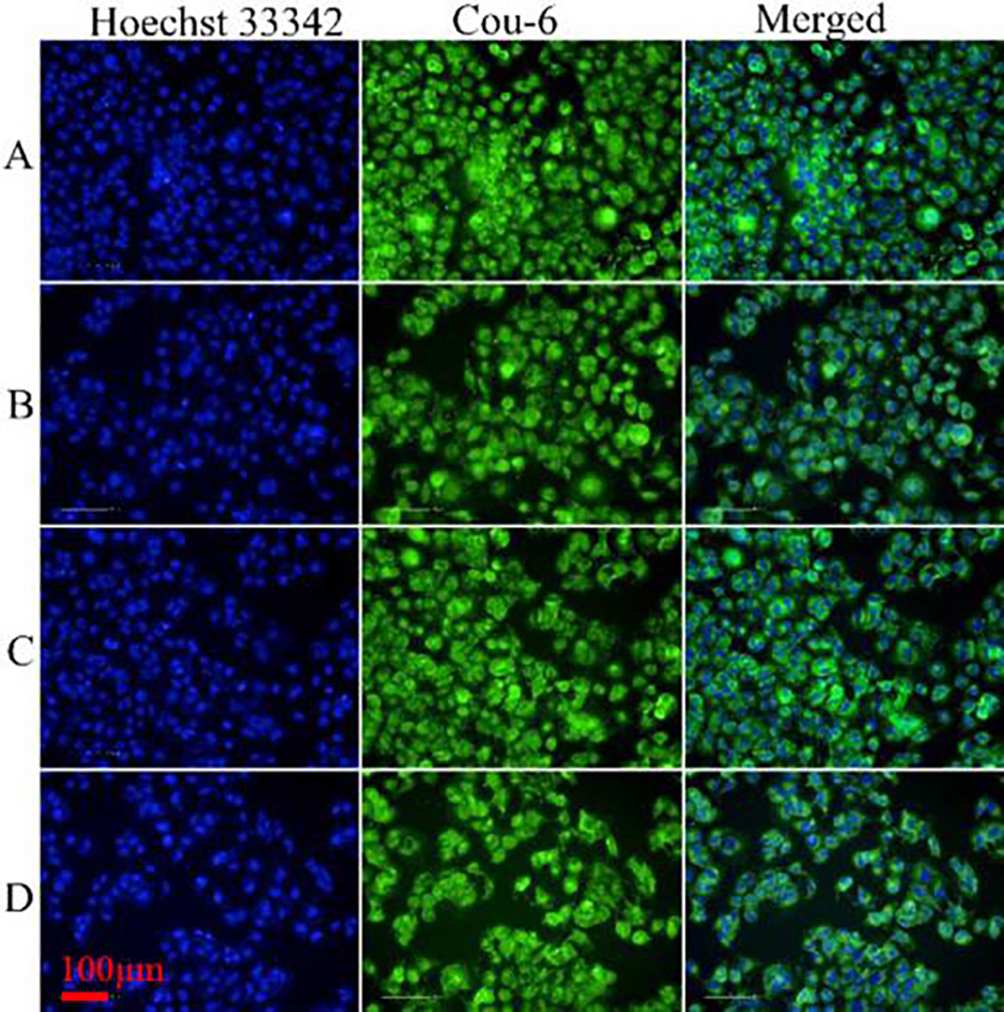


e


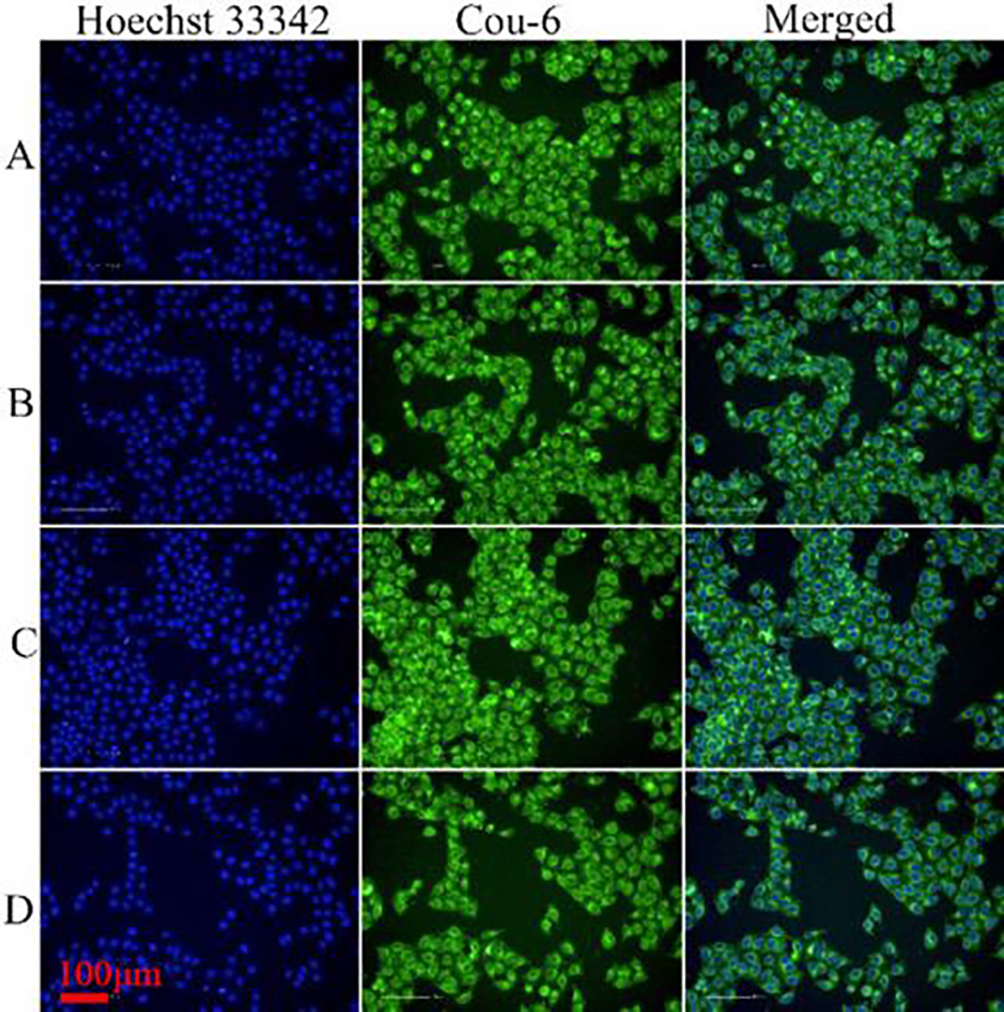


f


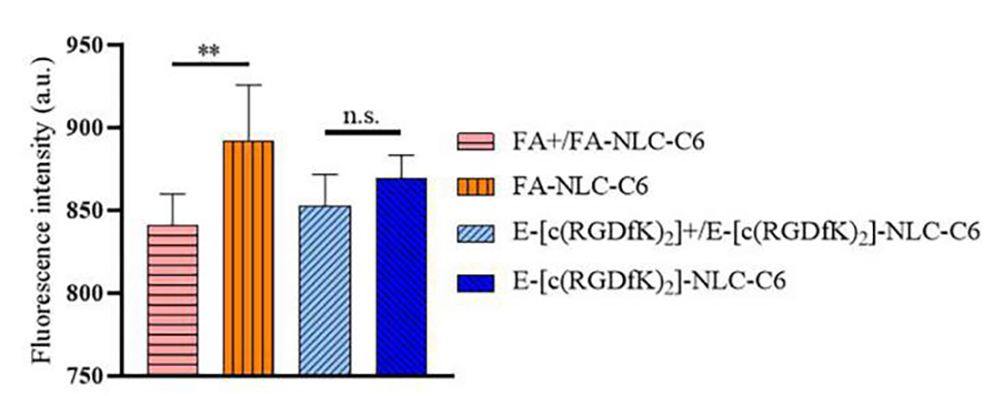


g


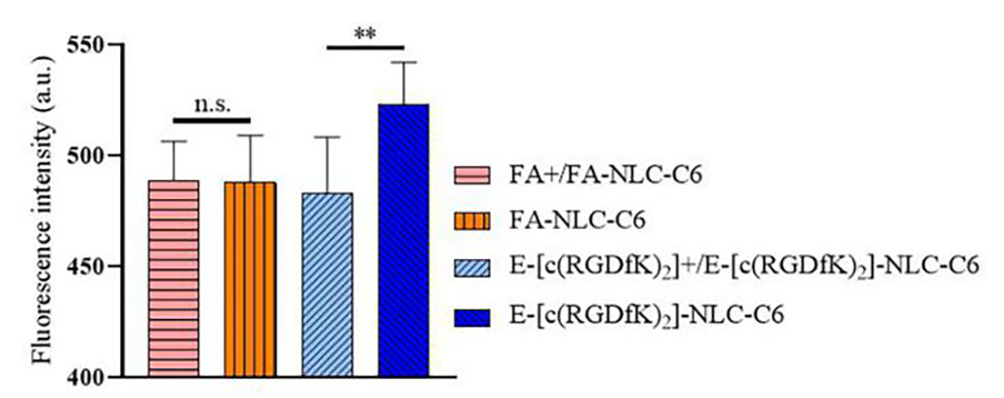


h

Figure S3. Fluorescent photos of four tumor cells in the cell uptake test after the receptor saturated treatment. Fluorescence picture (a) and intensity (c) of 4T1 cells, fluorescence picture (b) and intensity (d) of MDA-MB-231 cells, fluorescence picture (e) and intensity (g) of MCF-7 cells, fluorescence picture (f) and intensity (h) of A549 cells. A: FA+/FA-NLC-C6, B: FA-NLC-C6, C: E-[c(RGDfK)_2_]+/ E-[c(RGDfK)_2_]-NLC-C6, D: E-[c(RGDfK)_2_]-NLC-C6. ***p*<0.01. Results are expressed as mean ± SD, n=3.


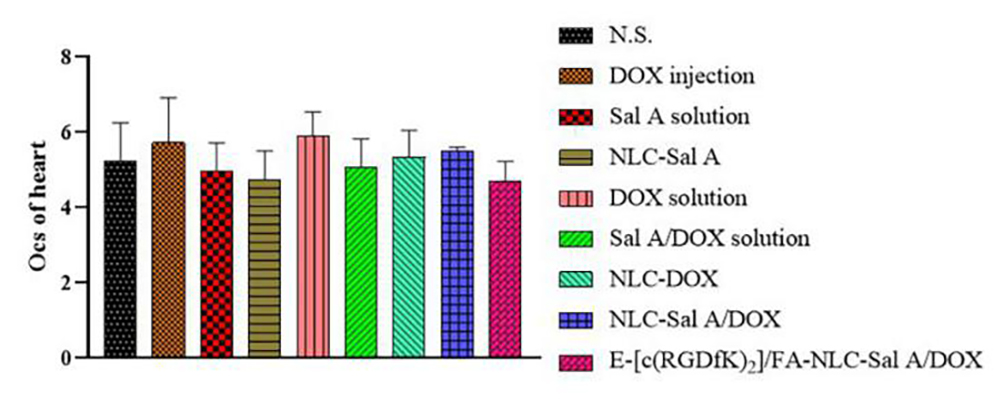


a


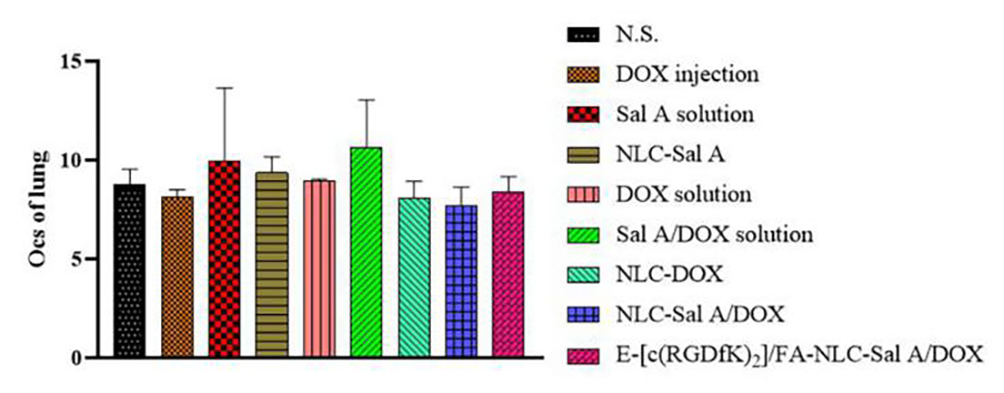


b


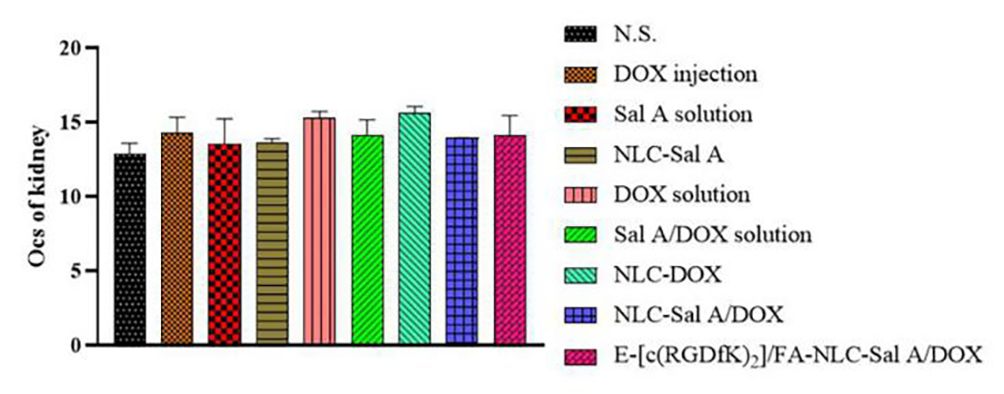


c


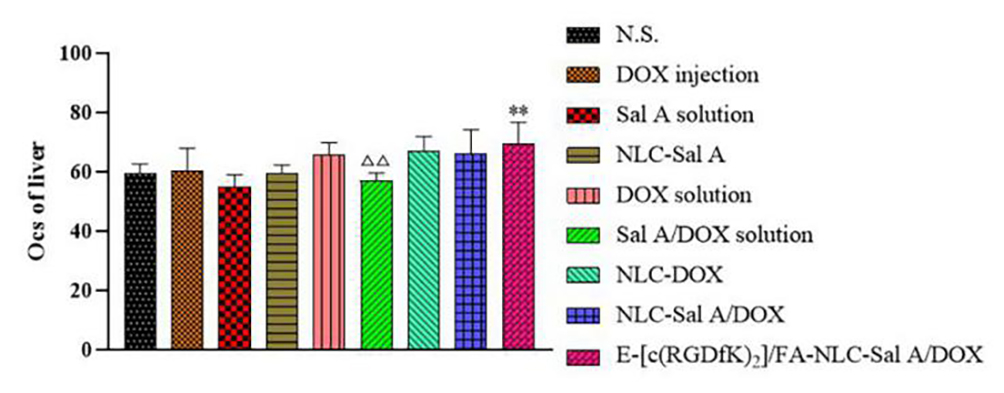


d


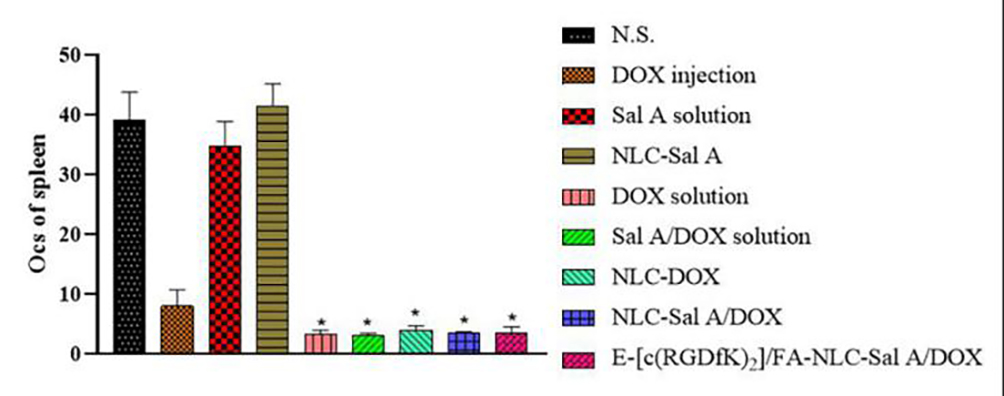


e

Figure S4. The Ocs of the isolated heart (a), lung (b), kidney (c), liver (d) and spleen (e) from the tumor-bearing female BALB/c mice treated after being treated with different preparations 12 days. **p<*0.05 vs DOX injection, ***p<*0.01 vs DOX injection, ^△△^*p<*0.01 vs DOX solution. Results are expressed as mean ± SD, n=6.
