# Supplementary material for: The anti-tumor and renoprotection study of E-[c(RGDfK)2]/folic acid co-modified nanostructured lipid carrier loaded with doxorubicin hydrochloride/salvianolic acid A
Source: J Nanobiotechnology. 2022 Sep 24;20:425. doi: 10.1186/s12951-022-01628-x (PMC9509648; doi:10.1186/s12951-022-01628-x)
Supplement: Supplementary file 2 — Additional file 2: Table S1. The IRV and IRw of the isolated tumor tissues excised from the tumor-bearing female BALB/c mice treated with different preparations. [file 12951_2022_1628_MOESM2_ESM.docx]

Table S1. The IRV and IRw of the isolated tumor tissues excised from the tumor-bearing female BALB/c mice treated with different preparations.

| Groups | IR_V_(%) | IR_W_(%) |
| --- | --- | --- |
| N.S | - | - |
| DOX injection | 51.84 | 51.31 |
| Sal A solution | 17.70 | 15.10 |
| NLC-Sal A | 24.06 | 27.00 |
| DOX solution | 49.25 | 37.68 |
| Sal A/DOX solution | 60.20 | 55.58 |
| NLC-DOX | 76.90 | 67.92 |
| NLC-Sal A/DOX | 85.09 | 78.89 |
| E-[c(RGDfK)_2_]/FA-NLC-Sal A/DOX | 90.72 | 83.94 |
